# A new yeti crab phylogeny: Vent origins with indications of regional extinction in the East Pacific

**DOI:** 10.1371/journal.pone.0194696

**Published:** 2018-03-16

**Authors:** Christopher Nicolai Roterman, Won-Kyung Lee, Xinming Liu, Rongcheng Lin, Xinzheng Li, Yong-Jin Won

**Affiliations:** 1 Department of Zoology, University of Oxford, Oxford, Oxfordshire, United Kingdom; 2 Department of Life Science, Division of EcoScience, Ewha Womans University, Seoul, Republic of Korea; 3 Deep-sea and Seabed Mineral Resources Research Center, Korea Institute of Ocean Science & Technology, Ansan, Republic of Korea; 4 Guangxi Academy of Oceanography, Nanning, China; 5 Institute of Oceanology, Chinese Academy of Science, Qingdao, China; 6 Third Institute of Oceanography, State Oceanic Administration, Xiamen, China; 7 University of Chinese Academy of Sciences, Beijing, China; 8 Laboratory for Marine Biology and Biotechnology, Qingdao National Laboratory for Marine Science and Technology, Qingdao, China; University of California, UNITED STATES

## Abstract

The recent discovery of two new species of kiwaid squat lobsters on hydrothermal vents in the Pacific Ocean and in the Pacific sector of the Southern Ocean has prompted a re-analysis of Kiwaid biogeographical history. Using a larger alignment with more fossil calibrated nodes than previously, we consider the precise relationship between Kiwaidae, Chirostylidae and Eumunididae within Chirostyloidea (Decapoda: Anomura) to be still unresolved at present. Additionally, the placement of both new species within a new “*Bristly*” clade along with the seep-associated *Kiwa puravida* is most parsimoniously interpreted as supporting a vent origin for the family, rather than a seep-to-vent progression. Fossil-calibrated divergence analysis indicates an origin for the clade around the Eocene-Oligocene boundary in the eastern Pacific ~33–38 Ma, coincident with a lowering of bottom temperatures and increased ventilation in the Pacific deep sea. Likewise, the mid-Miocene (~10–16 Ma) rapid radiation of the new *Bristly* clade also coincides with a similar cooling event in the tropical East Pacific. The distribution, diversity, tree topology and divergence timing of Kiwaidae in the East Pacific is most consistent with a pattern of extinctions, recolonisations and radiations along fast-spreading ridges in this region and may have been punctuated by large-scale fluctuations in deep-water ventilation and temperature during the Cenozoic; further affecting the viability of Kiwaidae populations along portions of mid-ocean ridge.

## Introduction

### Stable refugia?

The discovery in 1976 of deep-sea hydrothermal vents [1,2] hosting creatures morphologically very different from other deep-sea fauna, e.g. vestimentiferan tube worms [3] or exhibiting “primitive” characteristics, e.g. neolepadine stalked barnacles [4] led to an expectation that these taxa and those at cognate ecosystems might be representatives of ancient lineages hitherto unaffected by marine extinction events [5–7]. Subsequent paleontological and molecular evidence indicates, however, that the current diversity of most chemosynthetically-associated taxa is the consequence of Cenozoic radiations occurring after the Palaeocene/Eocene Thermal Maximum (PETM) ~55 million years ago (Ma); a warm period with widespread anoxia/suboxia in the world’s deep ocean basins [8,9]. Rather than deep-sea chemosynthetic ecosystems being stable refugia, the reverse may be the case. Invertebrates occupy narrow redox zones potentially close to their physiological limits and are largely reliant on chemolithoautotrophic bacteria as a single food source. This could leave them vulnerable to falls in ambient deep-water oxygen levels, thereby reducing the size of the redox zones that can be occupied and also the availability of food [9].

### Seep to vent

Related to this idea of vulnerability at hydrothermal vents is the notion that shallower, possibly temporally more stable, hydrocarbon seeps and organic food falls on continental margins may be an important source of vent diversity over evolutionary time [10]. Evidence for this at present is somewhat equivocal, though. Seep-associated siboglinids appear phylogenetically basal to vent-endemic taxa [11,12], consistent with this hypothesis, but what was once considered a similar pattern in mytilid mussels, with a shallow seep to deeper vent progression [13] now appears more complicated. More comprehensive taxonomic sampling reveals evidence of multiple radiations into both seeps and vent habitats by mussels inhabiting sunken organic substrates; although an evolutionary trend towards greater depth still exists [14–16]. Conversely, a recent phylogenetic study on chemosynthetic vesicomyid clams shows no clear shallow-to-deep evolutionary progression, with the basal *Vesicomya* genus found deeper than most other taxa within the group [17].

**Fig 1:**
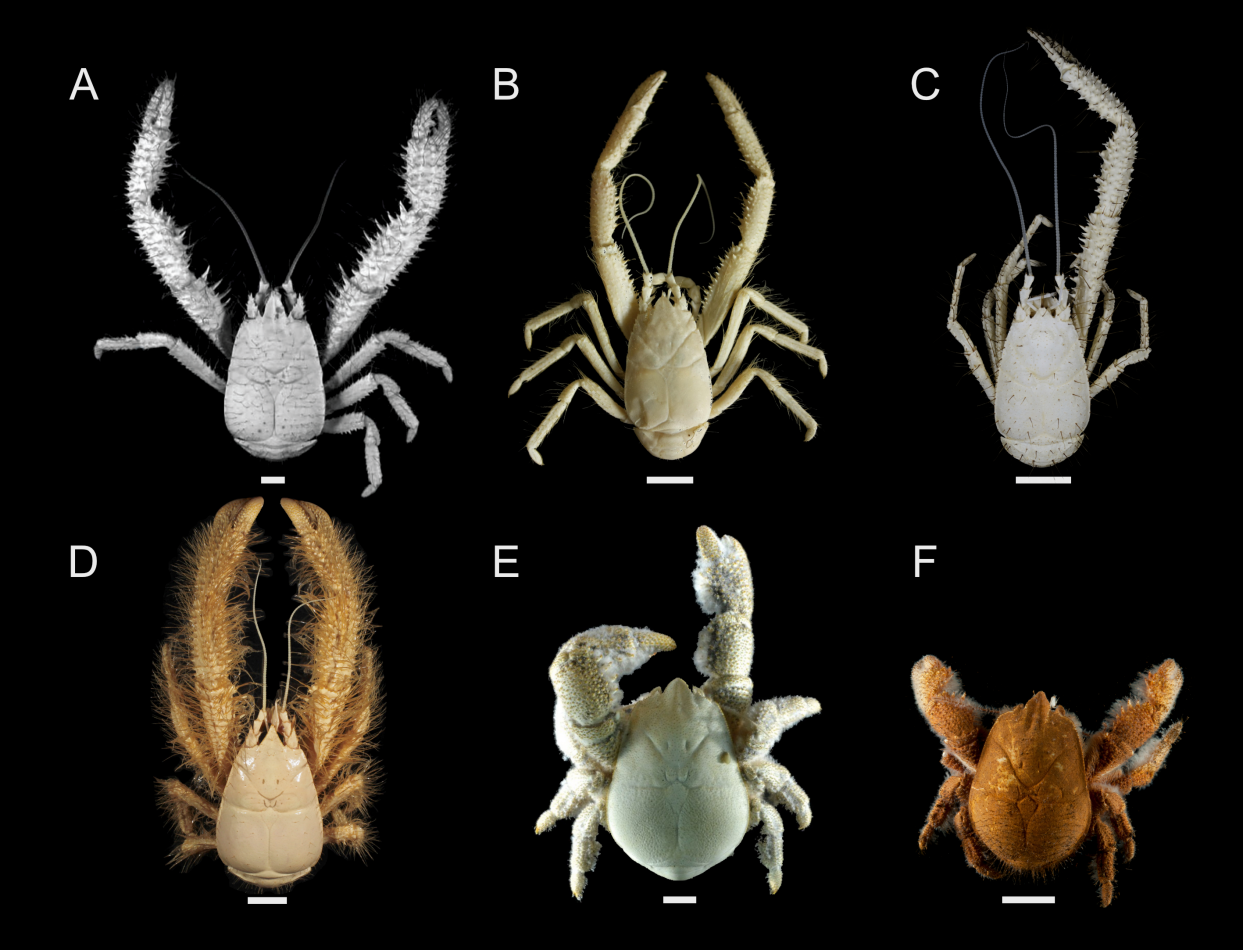
Photographs of known kiwaid squat lobsters (“yeti crabs”). A) *Kiwa puravida* modified from Thurber *et al*. [19] (CC BY 4.0); B) *Kiwa* sp. Galapagos Microplate; C) *Kiwa araonae* [28]; D) *Kiwa hirsuta* modified from Muséum National D’Histoire Naturelle (MNHN) crustacean collection–credit Noémy Mollaret (CC BY 4.0); E) *Kiwa tyleri* modified from Thatje *et al*. [23] (CC BY 4.0); (F) *Kiwa* sp. SWIR courtesy of David Shale. Scale bars are approximate and represent 10 mm.

**Fig 2:**
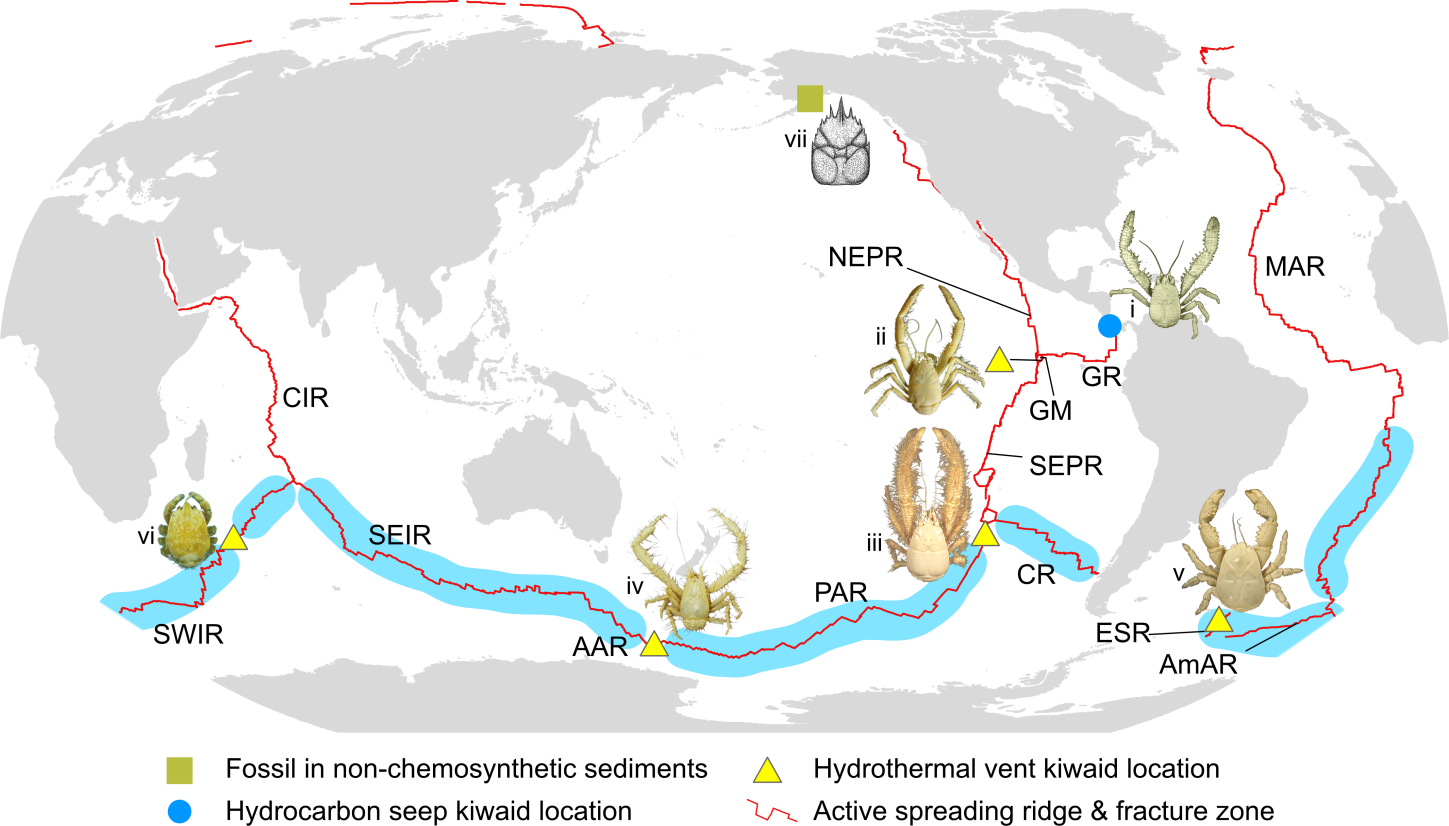
Map showing locations of kiwaids and the Cretaceous stem lineage fossil *Pristinaspina gelasina* in relation to land-masses and mid-ocean ridges. Kiwaid representations are: i) *Kiwa puravida* ii) *Kiwa* sp. GM, iii) *Kiwa hirsuta*, iv) *Kiwa araonae* v) *Kiwa tyleri* vi) *Kiwa* sp. SWIR. Land shapes and ridge positions are modified from the InterRidge Vents Database 2.1 static map (http://vents-data.interridge.org/maps). Areas of mid-ocean ridge in light blue denote unexplored regions that may support Kiwaidae. Spreading ridge abbreviations are as follows: NEPR = Northern East Pacific Rise; SEPR = Southern East Pacific Rise; GR = Galapagos Rift; GM = Galapagos Microplate; PAR = Pacific-Antarctic Ridge; AAR = Australian-Antarctic Ridge; CR = Chile Rise; ESR = East Scotia Ridge; AmAR = American-Antarctic Ridge; SWIR = Southwest Indian Ridge; CIR = Central Indian Ridge; SEIR = Southeast Indian Ridge; MAR = Mid-Atlantic Ridge. Photograph of *K*. *puravida* modified from Thurber *et al*. [19] (CC BY 4.0) and *Kiwa hirsuta* modified from Muséum National D’Histoire Naturelle (MNHN) crustacean collection–credit Noémy Mollaret (CC BY 4.0).

The recently discovered decapod crustacean family Kiwaidae [18] (Fig 1), which is found exclusively at deep-sea chemosynthetic ecosystems, has contributed to the discussion regarding the origin of vent endemic fauna. These anomuran squat lobsters in the superfamily Chirostyloidea, commonly known as “Yeti crabs”, appear to derive the bulk of their nutrition from chemosynthetic episymbiotic bacteria growing upon hair-like setae, which sprout from various parts of their ventral surface and appendages [18–23]. All kiwaids have been collected from hydrothermal vents, with the exception of *Kiwa puravida* which was collected from cold seeps on the Pacific continental slope near Costa Rica [19] (Fig 2). All species are located in the Pacific or in the Pacific sector of the Southern Ocean, with the exception of *Kiwa tyleri*, which was found in exceptionally high density at vents on the East Scotia Ridge (ESR) in the Atlantic sector of the Southern Ocean [24] and a similar undescribed species from the Southwest Indian Ridge (SWIR) in the Indian Ocean [25]. Roterman *et al*. [26] performed a nine-gene phylogenetic analysis on four kiwaid species with other chirostyloid squat lobsters, revealing support for an affinity between Kiwaidae and the coral-associated family Chirostylidae, having diverged from each other ~90–120 Ma; with Kiwaidae then radiating ~30 Ma. The timing of this radiation is consistent with the observation of Vrijenhoek *et al*. [9] that most chemosynthetically-associated clades radiated after the PETM during the Cenozoic. Within Kiwaidae, Roterman *et al*. [26] found a basal split between the seep-associated *K*. *puravida* and a vent endemic clade comprising *K*. *hirsuta* found on the Pacific-Antarctic Ridge (PAR), *K*. *tyleri* and *Kiwa* sp. SWIR, which they interpreted as consistent with a seep-to-vent evolutionary progression. Based on the Alaskan location of a Cretaceous stem lineage fossil, *Pristinaspina gelasina* [27] and the northern hemisphere location of *K*. *puravida*, a Northeast Pacific origin for the clade was inferred. Within the vent-associated clade the other two non-Pacific species appeared to have split from the *K*. *hirsuta* lineage ~20 Ma; likely accessing the Atlantic sector of the Southern Ocean from the Pacific via the recently-opened Drake Passage.

### Recent discoveries

Since this publication, two more species of kiwaid have been discovered. The first of these, *Kiwa* sp. GM, which remains undescribed, was collected from hydrothermal vents on the Galapagos Microplate, a distinct spreading system between the Galapagos Rift to the East, and the northern and southern portions of the East Pacific Rise (EPR) to the West [29] (Fig 2). The second species, *Kiwa araonae* [28], was collected from vents on the Australian-Antarctic Ridge (AAR) in the southwest Pacific sector of the Southern Ocean (Fig 2). The discovery of this species greatly extends the known range of Kiwaidae on mid-ocean ridges by ~6,500 km from the previous most westerly location of *Kiwa hirsuta* on the PAR and is the second species to be found in the Southern Ocean, after *K*. *tyleri*. Lee *et al*. [28] found a close affinity between *K*. *araonae* and the seep-associated *K*. *puravida* according to morphological examinations and molecular phylogenetic analyses using the 16S ribosomal RNA gene, despite a ~12,000 km distance between the two. Given that geographically, the nearest kiwaid to this species is *K*. *hirsuta*, Lee *et al*. [28] have suggested that the spread of kiwaids around the globe may have been more complicated than first assumed and have questioned the simple seep-to-vent evolutionary progression proposed by Roterman *et al*. [26].

### Aims

In light of these recent discoveries and the questions that they raise, we present a phylogeny to update that of Roterman *et al*. [26] by incorporating the new species. Furthermore, owing to the recent addition of new anomuran sequences from other studies, as well as new sequences generated herein, we are able to increase the number of fossil-calibrated nodes from 3 to 7 in the divergence analyses, thus refining the original divergence date estimates. Our principal aim is to gain new insights into the evolution of Kiwaidae and to discuss them in the context of recent findings regarding their distinctive behaviour and life history characteristics [23,30–32]. Finally, by synthesising the literature presently accrued concerning Kiwaidae, we aim to draw wider inferences about the evolution and resilience of deep-sea chemosynthetically-associated megafauna.

## Materials and methods

### Taxon sampling

In total, 34 OTUs were employed in the analyses, 23 of which featured in Roterman *et al*. [26]. This study incorporates all known kiwaid anomurans; two more than previously, bringing the total to six. This includes the Pacific-Antarctic Ridge vent-associated *Kiwa hirsuta* [18], the seep-associated *Kiwa puravida* from the Pacific continental slope off Costa Rica [19], *Kiwa tyleri* from vents on the East Scotia Ridge [23] and the recently discovered *Kiwa araonae* from vents on the Australian-Antarctic Ridge [28]. Two undescribed species are also included: one collected from vents on the Southwest Indian Ridge, *Kiwa* sp. SWIR [25] and another–*Kiwa* sp. GM–collected from the Galapagos Microplate at the triple junction between the Galapagos rift and the Northern and Southern East Pacific Rise. In addition, nine more non-kiwaid anomurans were included in this study (S1 Table).

### DNA extraction, PCR amplification and sequencing

Total genomic DNA was extracted from pereopods, pleopods or antennae using either Qiagen DNeasy Blood and Tissue Kit following manufacturer’s instructions or CTAB DNA extraction protocol [33]. Nine gene sequence regions were amplified: fragments of the ribosomal RNA genes 16S (~650 bp), 18S (~1900 bp) and 28S (~300 bp) as well as 350–650 bp fragments of the protein-coding genes cytochrome oxidase subunit 1 (COI), sodium potassium ATPase ⍺ subunit (NaK), enolase (En), arginine kinase (AK), glyceraldehyde 3-phosphate dehydrogenase (GAPDH) and phosphoenolpyruvate carboxykinase (PEPCK). Of these genes, two are mitochondrial (16S, COI). The primers used are listed in supporting information (S2 Table). PCR reaction and general amplification procedures varied with taxon and are covered in supporting information (S1 File). Thirty new gene fragments were generated in this study and have been deposited in GenBank (S1 Table). Additional sequence fragments were downloaded from GenBank, including fragments originally generated in Roterman *et al*. [26]; some of which were extended using original contigs with GenBank records updated accordingly.

### Alignments and partitioning

Protein-coding genes, (COI, NaK, En, AK, GAPDH and PEPCK) were aligned using the Geneious alignment tool in Geneious Pro 6.1.8 and ribosomal genes (16S, 28S, and 18S) were aligned using the online version of MAFFT 7.309 [34]. For all three ribosomal genes, the X-INSi framework [35] with the CONTRAfold algorithm [36] was used, which incorporates predicted secondary structure. Alignments were then adjusted by eye. Highly variable regions in the rRNA fragments that were hard to align were excised using Gblocks 0.91 [37], with the least stringent settings and all gap positions allowed (S3 Table). The final concatenated alignment, totalling 6,513 bp, is as follows: 16S (548 bp), 18S (1,875 bp), 28S (340 bp), COI (657 bp), NaK (663 bp), En (390 bp), AK (669 bp), GAPDH (732, bp), PEPCK (639 bp). PartitionFinder 2.1.1 [38] was used to determine the best partitioning scheme (for IQ-TREE, MrBayes and BEAST analyses) and the substitution model (for IQ-TREE, MrBayes) for each partition according to the corrected Akaike Information Criterion (AICc) and the Bayesian Information Criterion (BIC) with branch lengths linked and a greedy search algorithm. The optimal partitioning schemes are shown in supporting information (S4 Table).

### Phylogenetic analyses

**Fig 3:**
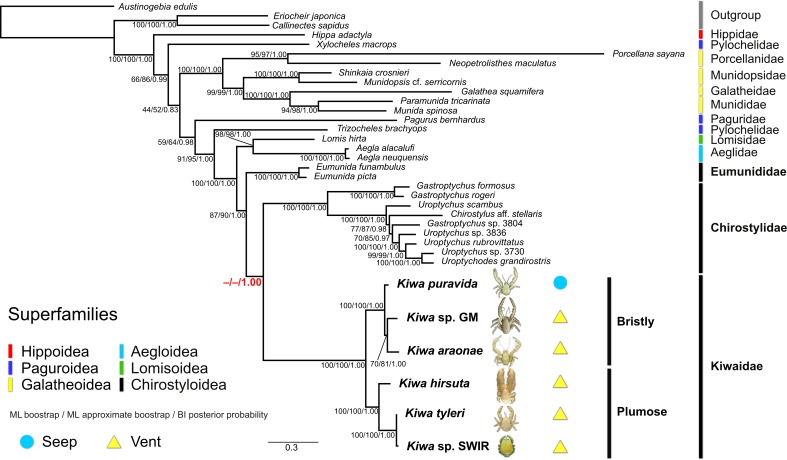
Bayesian tree topology of a 9-partition anomuran crustacean dataset generated in MrBayes 3.2.6. Node support numbers represent ML bootstrap and ML ultrafast approximate bootstrap percentages generated in IQ-TREE 1.5.4 and Bayesian posterior probabilities from MrBayes. Node support values in red bold highlight topology incongruence between the different phylogenetic approaches. Photographs of the six known kiwaids are superimposed next to their names (highlighted in bold). Photograph of *K*. *puravida* modified from Thurber *et al*. [19] (CC BY 4.0) and *Kiwa hirsuta* courtesy of IFREMER.

**Fig 4:**
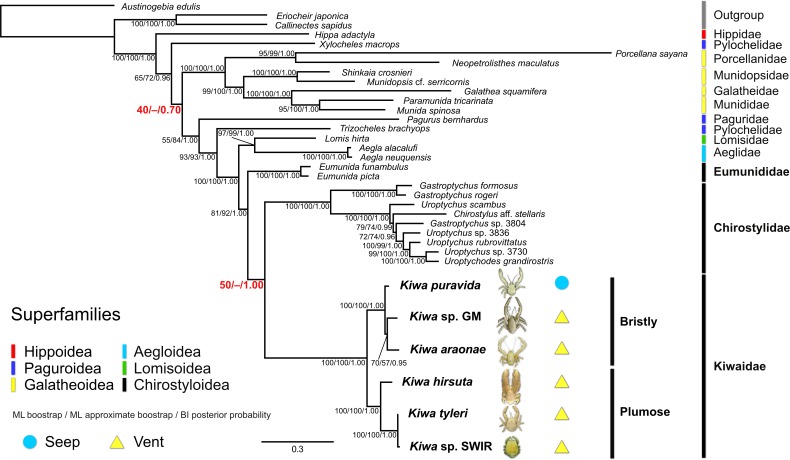
Bayesian tree topology of a 14-partition anomuran crustacean dataset generated in MrBayes 3.2.6. Node support numbers represent ML bootstrap and ML ultrafast approximate bootstrap percentages generated in IQ-TREE 1.5.4 and Bayesian posterior probabilities from MrBayes. Node support values in red bold highlight topology incongruence between the different phylogenetic approaches. Photographs of the six known kiwaids are superimposed next to their names (highlighted in bold). Photograph of *K*. *puravida* modified from Thurber *et al*. [19] (CC BY 4.0) and *Kiwa hirsuta* courtesy of IFREMER.

Two different methods for determining phylogenies were performed in this study: maximum likelihood (ML) and Bayesian inference (BI). ML analyses were performed using IQ-TREE 1.5.4 [39] both with 1000 non-parametric bootstrap replicates and also with 100,000 “ultrafast bootstrap approximation” replicates; a method that is less conservatively biased than standard bootstrapping [40]. BI was performed using MrBayes 3.2.6 [41] on the CIPRES computing cluster [42]. Metropolis coupled Monte Carlo Markov Chains (MCMC) were run for 100 million generations in two simultaneous runs, each with four differently heated chains. Convergence of the analyses was validated by the standard deviation of the split frequencies and by monitoring of the likelihood values using Tracer 1.6 [43]. Topologies were sampled every 1000 generations, with the first 25% of trees discarded as burn-in. The resulting BI topologies are illustrated (Figs 3 and 4) with bootstrap and approximate bootstrap support values from the ML analyses included. ML topologies are in supporting information (S1–S4 Figs).

### Divergence estimation using fossil calibration

Bayesian estimation of fossil-calibrated divergence times were performed with BEAST 2.4.3 [44] on the entire concatenated dataset using the CIPRES computing cluster. The same partition schemes (both AICc and BIC determined) used in the phylogenetic analyses were employed, with substitution models unlinked across all partitions. For each partition scheme, analyses were implemented with clock models linked and unlinked across all partitions. To explore the effect of chirostyloid tree topology on Kiwaidae divergence estimates, two topology constraints were imposed, both based on the MrBayes analyses: the first with a monophyletic Kiwaidae-Chirostylidae clade (K-C) within Chirostyloidea and the second with a monophyletic Kiwaidae-Eumunididae clade (K-E). Consequently, eight different BEAST analyses were performed. To avoid over parameterisation of pre-determined substitution models leading to poor mixing of the MCMC chain, the bModelTest module was used on the PartitionFinder partition scheme [45] with the default transition-transversion split option. In bModelTest, substitution models are determined during the actual MCMC analysis using a Bayesian approach, by allowing substitution models to switch, resulting in better MCMC mixing. A relaxed lognormal clock was used with a Yule speciation model, after preliminary runs revealed no discernible difference between Yule and Birth-Death speciation. Two independent runs per analysis were performed for 200 million generations and sampled every 100,000 generations with 10% of samples removed as burn-in. Runs were combined using LogCombiner 2.4.6. BEAST output was visualised on Tracer 1.6. Discrete ancestral trait analyses [46] were performed in the divergence BEAST runs to infer the likely habitat association of ancestral kiwaids and other chirostyloids. Operational taxonomic units (OTUs) were assigned to one of four habitat types based on published data, or observations during specimen collection (S5 Table). The four categories were “Vent”, “Seep”, “Coral” or “Other”. Missing data (“?”) was included as a fifth state.

### Fossil dating methodology

Fossils have been chosen that represent the oldest known member of a particular clade at the time of writing. The assignments of these fossils to specific clades is based on interpretations in the published literature. The fossil calibration methodology, including the placement of fossils at crown and stem nodes follows the suggestions of Parham *et al*. [47]. The most recent possible age for a given fossil was used as a minimum “hard” age constraint for a given node on the tree, with the maximum age being “soft” and liberal to account for the fact that the fossil is almost certainly younger than the “true” age of the node it is being used to calibrate. An exponential, rather than a normal or lognormal prior distribution shape, with the highest probability assigned to the minimum fossil age, was used in BEAST owing to the absence of paleontological information regarding the placement of the probability distribution mode, as suggested by Ho & Phillips [48]. Consequently, the date ranges estimated in this study represent the likely minimum (youngest) possible constraints for the ages of the clades in interest, based on the current fossil record. The soft upper bounds (95% quantile) of the prior distribution i.e. the maximum bounds for the fossil calibrations, match the oldest possible geochronological dates for fossils representative of proximate higher taxonomic levels as per Ho & Phillips [48]. Fossils were assigned ages based on the current ICS International Chronostratigraphic Chart dating scheme for the particular stratum in which they were reported to be found in the original literature [49]. Justifications for fossil placements at nodes are listed below.

### Calibration scheme

1. The fossil stem-lineage anomuran, *Platykotta akaina* [50], of the Upper Triassic (Norian-Rhaetian, 201.3–227 Ma) was chosen as a minimum constraint (201.3 Ma) for the divergence between Anomura and Brachyura, which recent phylogenetic studies suggest are sister groups [51–53]. Recent phylogenies have tended to place the former infraorder Thalassinidea (mud and ghost shrimps), or the two infraorders into which it has been split, Gebiidea and Axiidea, as basal to Anomura and Brachyura [51–56], or in the case of Tsang *et al*. [57], just Gebiidea as basal. Therefore *Upogebia obscura* [58], attributed to Gebiidea [59] from the early Triassic Bunter sandstone (242–252.9 Ma), has been chosen as the soft 95% upper bound (252.9 Ma).
2. The oldest fossil hermit crab (Paguroidea: Schobertellidae), *Schobertella hoelderi* [60], from the Lower Jurassic (Hettangian, 199.3–201.3 Ma) was chosen as the minimum age constraint for the divergence of hippoid and non-hippoid anomurans. The five-gene molecular phylogeny of Tsang *et al*. [61] as well as that of Bracken-Grissom *et al*. [52] using a different five gene dataset, revealed the non-hippoid anomurans to be split into two groups: one comprising Galatheoidea and some pylochelid (subfamily Pylocheninae) symmetrical hermit crabs and the other comprising Aegloidea, Lomisoidea, Chirostyloidea, other pylochelids (subfamily Trizochelinae) and the asymmetrical hermit crabs (including Lithodoidea). Both groupings featured paguroid hermit crabs as basal. Given this, the early appearance of a paguroid hermit crab in the anomuran fossil record is unsurprising and we consider the use of *S*. *hoelderi* as a minimum constraint for the divergence of hippoid and non-hippoid anomurans is appropriate. The soft 95% upper bound was set to *P*. *akaina* (227 Ma).
3. The oldest known fossil for a munidopsid squat lobster, *Palaeomunidopsis moutieri* [62], from the Middle Jurassic (Bathonian, 166.1–168.3 Ma) was chosen as a minimum constraint for the divergence between Munidopsidae and other non-porcellanid galatheoids. The soft 95% upper bound was set to *S*. *hoelderi* (201.3 Ma).
4. The oldest munidid squat lobster, *Juracrista perculta*, from the Upper Jurassic (Tithonian, 145–151.9 Ma) [63] was chosen as the minimum constraint for the divergence between Munididae and Galatheidae. The soft 95% upper bound was set to *P*. *moutieri* (168.3 Ma).
5. The oldest pylochelid hermit crab in the subfamily Trizochelinae, *Ammopylocheles robertboreki*, from the Upper Jurassic (Oxfordian, 157.3–163.5 Ma) [64] was chosen as the minimum constraint for the divergence between the trizocheline hermit crab *Trizocheles brachyops* and the superfamilies Lomisoidea, Aegloidea and Chirostyloidea. Both Bracken-Grissom *et al*. [52] and Tsang *et al*. [61] revealed the polyphyly of the pylochelid symmetrical hermit crabs, with the Trizochelinae as basal within a clade comprising Lomisoidea, Aegloidea and Chirostyloidea (and also Parapaguridae in the case of Tsang *et al*. [61]. The soft 95% upper bound was set to *S*. *hoelderi* (201.3 Ma).
6. The oldest aegloid squat lobster, *Protaegla miniscula* [65], from the Lower Cretaceous (Albian, 100.5–113 Ma) was used as the minimum age constraint for the divergence between Aegloidea and Lomisoidea. The soft 95% upper bound was set to *A*. *robertboreki* (163.5 Ma).
7. *Pristinaspina gelasina* dated to the Upper Cretaceous (66–100.5 Ma) has been identified as a likely stem-lineage kiwaid [26,66] and has been incorporated accordingly, with the soft 95% upper bound set to *A*. *robertboreki* (163.5 Ma).

## Results

### Data summary

Of the 34 sequence sets in this study, 20 were complete, 6 were missing a single gene fragment and 8 were missing 2–4 fragments. A total of 30 new DNA sequences were generated with 124 existing GenBank sequences from Roterman *et al*. [26] being updated. PartitionFinder yielded a 9- and a 14-partitioned dataset using BIC and AICc respectively. Both schemes treated the ribosomal genes each as separate partitions, with the protein-coding genes split into partitions of various sizes containing either 1st, 2nd or 3rd codon positions (S4 Table). Phylogenetic trees were generated in MrBayes and IQ-TREE. The MrBayes generated Bayesian inference tree topologies with posterior probability values for the two partition schemes are presented here, with ML bootstrap and ultrafast approximate bootstrap support percentages generated in IQ-TREE also included at congruent nodes. Separate ML trees generated in IQ-TREE are included in supporting information (S1–S4 Figs).

### Tree topology

BI and ML analyses produced similar, but slightly different results for both partition schemes (BI topologies including ML bootstrap support values, Figs 3 and 4; ML topologies, S1–S4 Figs). All analyses strongly supported the monophyly of a clade comprising Aegloidea, Lomisoidea and Chirostyloidea (ML bootstrap = 100%, ML approximate bootstrap = 100%, BI posterior probability = 1.00). Support for the monophyly of Chirostyloidea containing Kiwaidae, Eumunididae and Chirostylidae was also good (ML bootstrap = 81–87%, ML approximate bootstrap = 90–92%, BI posterior probability = 1.00). Within Chirostyloidea, BI analyses strongly supported the monophyly of Kiwaidae-Chirostylidae, with Eumunididae as basal (posterior probability = 1.00), but ML analyses yielded equivocal results (highlighted in red bold, Figs 3 and 4). For the 14-partition scheme (Fig 4), there was equal ML bootstrap support for a Kiwaidae-Chirostylidae clade and a Kiwaidae-Eumunididae clade (50%), but not with the 9-partition scheme (Fig 3) where there was slightly stronger bootstrap support for a Kiwaidae-Eumunididae clade (53%, see S1 Fig). With the ultrafast bootstrap approximation approach, both partition schemes resulted in support for a Kiwaidae-Eumunididae clade (56% for the 9-partition scheme and 97% for the 14-partition scheme, S3 and S4 Figs respectively). Within Kiwaidae, all analyses resulted in the same tree topology, comprising two clades: one containing *Kiwa puravida* as basal to a *Kiwa* sp. GM and *Kiwa araonae* clade, and another comprising *Kiwa hirsuta* as basal, with *Kiwa tyleri* and *Kiwa* sp. SWIR as sister taxa. Support for all nodes within Kiwaidae was very strong (ML bootstrap = 100%, ML approximate bootstrap = 100%, BI posterior probability = 1.00), with the exception of the *Kiwa* sp. GM*-K*. *araonae* clade, where support was weaker (ML bootstrap = 70%, ML approximate bootstrap = 57–81%, BI posterior probability = 0.95–1.00). For ease of reporting, the *K*. *puravida-K*. *araonae-Kiwa* sp. GM clade will be referred to as *Bristly* Kiwaidae, owing to the conspicuous covering of more coarse, bristly setae, as compared to the clade comprising *K*. *hirsuta*, *K*. *tyleri* and *Kiwa* sp. SWIR, which will be referred to as *Plumose* Kiwaidae, owing to the noticeably higher density of plumose setae over their bodies (Fig 1).

### Divergence dating and ancestral habitats

**Fig 5:**
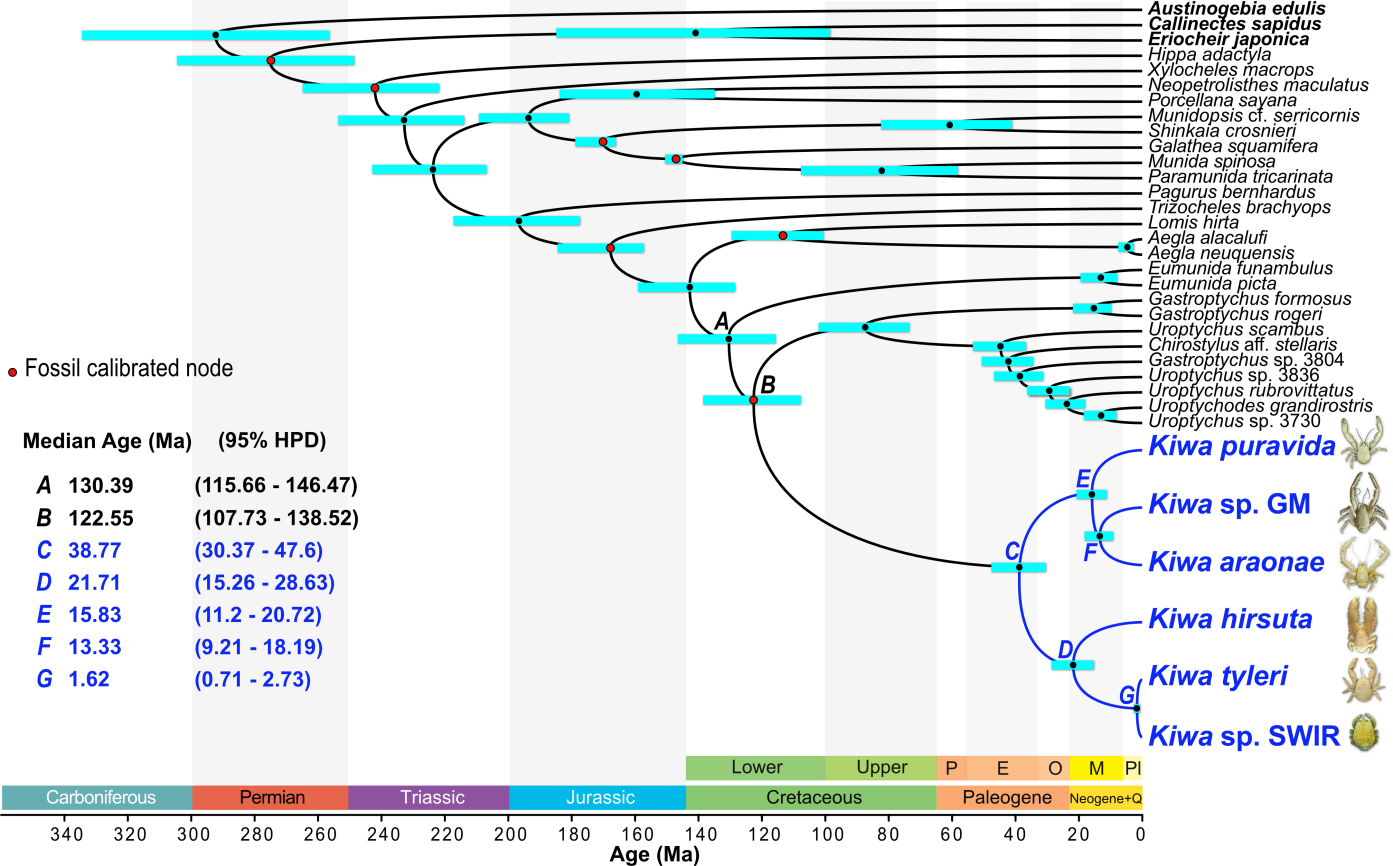
Divergence time estimates for a 9-partition anomuran crustacean dataset with a monophyletic Kiwaidae-Chirostylidae clade topological constraint as calculated with a relaxed, unlinked lognormal clock on BEAST 2.4.3. Node bars represent the 95% highest posterior density (HPD) interval for nodal age. Nodes marked by letters show dates of interest to this study. Dates highlighted in blue are of particular interest. Nodes marked with a red spot are fossil calibrated. Geological periods and epochs are shown at the bottom: P = Palaeocene; E = Eocene; O = Oligocene; M = Miocene; Pl = Plio-Pleistocene; Q = Quaternary. Geological colours correspond to those of the International Chronostratigraphic Chart [49], which conforms to the Commission for the Geological Map of the World (http://www.ccgm.org). Photograph of *K*. *puravida* modified from Thurber *et al*. [19] (CC BY 4.0) and *Kiwa hirsuta* courtesy of IFREMER.

**Fig 6:**
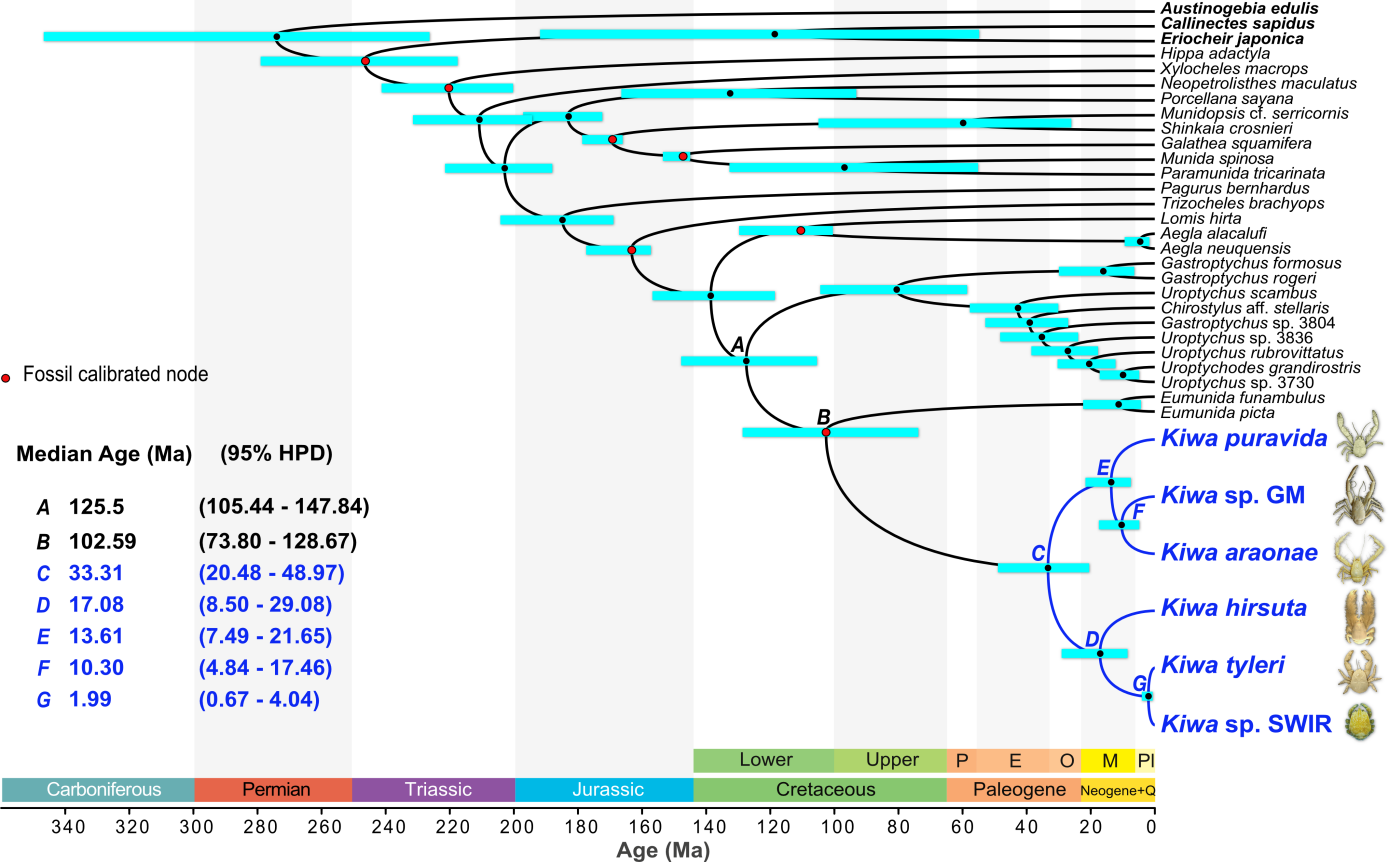
Divergence time estimates for a 14-partition anomuran crustacean dataset with a monophyletic Kiwaidae-Eumunididae clade topological constraint as calculated with a relaxed, lognormal clock linked across partitions on BEAST 2.4.3. Node bars represent the 95% highest posterior density (HPD) interval for nodal age. Nodes marked by letters show dates of interest to this study. Dates highlighted in blue are of particular interest. Nodes marked with a red spot are fossil calibrated. Geological periods and epochs are shown at the bottom: P = Palaeocene; E = Eocene; O = Oligocene; M = Miocene; Pl = Plio-Pleistocene; Q = Quaternary. Geological colours correspond to those of the International Chronostratigraphic Chart [49], which conforms to the Commission for the Geological Map of the World (http://www.ccgm.org). Photograph of *K*. *puravida* modified from Thurber *et al*. [19] (CC BY 4.0) and *Kiwa hirsuta* courtesy of IFREMER.

Eight different fossil-calibrated divergence and ancestral trait analyses were performed in BEAST, and key node ages and habitat association probabilities are reported in supporting information (S6 Table). All divergence estimates across the analyses were broadly in agreement, but the largest difference in age estimates were between analyses with a linked molecular clock across all partitions and those that were unlinked across partitions; with unlinked analyses producing older node estimates. For example, in linked analyses, the median estimates for the most recent common ancestor (MRCA) of Chirostyloidea ranged from 125.8–127.5 million years ago (Ma) (95% Highest Posterior Density–HPD–range of 103.4–147.8 Ma), whereas in unlinked analyses the median estimates were 130.4–134.1 Ma (95% HPD of 115.7–150.6 Ma). Changing the number of partitions had little effect and changing the tree topology of the three chirostyloid families only affected the MRCA age for the chirostyloid sister taxa; with the hypothetical Kiwaidae-Eumunididae clade MRCA being generally younger (median age of 102.7–111 Ma) than the MRCA age for the Kiwaidae-Chirostylidae clade (median age of 108.4–125.2 Ma). To illustrate this range of estimates, two divergence trees are presented here: a 9-partition scheme with unlinked clocks for each partition, showing older ages with narrower 95% HPD intervals (Fig 5), and a 14-partition scheme with a linked clock across partitions, showing generally younger ages with wider 95% HPD intervals (Fig 6). Across all eight analyses, the median age estimates for the MRCA of Kiwaidae are 33.2–38.8 Ma (95% HPD ranges of 20.5–49.7 Ma). Within Kiwaidae, median MRCA age estimates for *Plumose* Kiwaidae are 17.1–21.71 Ma (95% HPD range of 8.3–29.4 Ma) and the median MRCA age estimates for *Bristly* Kiwaidae are 13.3–15.9 Ma (95% HPD ranges of 7–21.8 Ma). Within *Bristly* Kiwaidae, the median estimates for the divergence between *K*. *araonae* and *Kiwa* sp. GM are 10.1–13.5 Ma (95% HPD range of 4.8–18.3 Ma), and within *Plumose* Kiwaidae, the divergence estimates between *K*. *tyleri* and *Kiwa* sp. SWIR are 1.5–2.1 Ma (95% HPD range of 0.7–4.3 Ma).

Discrete trait reconstructions of the habitat affinity of kiwaid ancestors were consistent across the 8 different BEAST analyses (S6 Table). The probabilities of the common ancestor of Kiwaidae being chemosynthetically associated are high (98.3–98.5%), with 95.2–96.0% probabilities for vent association and 2.6–3.1% for seep association. For the common ancestor of *Plumose* Kiwaidae, probabilities of vent association are also high (98.5–98.7%) and for the common ancestor of *Bristly* Kiwaidae (the ancestor of the seep-associated *K*. *puravida*) probabilities of vent association are similarly so (95.4–96.4%). Conversely, the probabilities of the common ancestor of Kiwaidae and its sister taxon being chemosynthetically associated are 13.2–20.2%, with the largest share of probabilities going to coral association (72.6–81.2%). For the common ancestor of all Chirostyloidea, the probabilities of chemosynthetic association are 11.8–12.8%, with coral association making up 67.9–75.0% and association with other habitats accounting for 13.2–19.8%.

## Discussion

### Chirostyloid topology

This study amounts to an expansion of Roterman *et al*. [26], with two more kiwaids and nine more non-kiwaids, a longer overall alignment of the nine-gene dataset (6,513 bp vs 5,560 bp) and seven fossil-calibrated nodes as opposed to three. Broadly, the two different partition schemes of the sequence data and the different phylogenetic approaches employed produced very similar tree topologies, both in relation to each other and compared with the tree generated by Roterman *et al*. [26]. In all cases a clade was resolved comprising the superfamilies Chirostyloidea (Kiwaidae, Chirostylidae and Eumunididae), Aegloidea and Lomisoidea (Figs 3 and 4) in keeping with previous research [26,52,61,67]. This study and the gene-only analyses of Bracken-Grissom *et al*. [52] placed a pylochelid (symmetrical) hermit crab of the subfamily Trizochelinae as basal to this group. Consequently, the symmetrical hermit crab family Pylochelidae appears to be polyphyletic and that the common ancestor of Chirostyloidea-Aegloidea-Lomisoidea may have been a symmetrical hermit crab; as proposed by Tsang *et al*. [61]. Within Chirostyloidea, however, the relationship between Kiwaidae and the deep-water coral-associated squat lobsters Chirostylidae and Eumunididae appears more ambiguous. Roterman *et al*. [26] found Kiwaidae and Chirostylidae to be sister families, with Eumunididae as basal with strong Bayesian posterior probability support for Kiwaidae-Chirostylidae (> 0.99), but weaker maximum likelihood (ML) bootstrap support (77%). They noted that Kiwaidae and Chirostylidae both produced similar, large, lecithotrophic larvae [30,68] as opposed to Eumunididae, which produce smaller, more numerous eggs [69]. In the analyses presented here, only Bayesian tree topologies and the ML analysis for the 14-partition scheme produced a Kiwaidae-Chirostylidae clade over Kiwaidae-Eumunididae. Consequently, we consider the matter regarding the interfamilial relationships within Chirostyloidea to be unresolved for the time being. More comprehensive taxon sampling, particularly within Eumunididae, along with either more gene fragments or perhaps much larger genomic datasets may be required to clarify the phylogenetic relationships between the families within Chirostyloidea.

### Kiwaid topology, geographical and habitat origins

Within Kiwaidae, strong support for most of the nodes across all partitions and analyses engenders confidence that the broad topology presented here is robust, with the *Plumose* clade topology the same as the previous analysis [26]. The major difference in this study is the addition of two species, closely related to *K*. *puravida* within a *Bristly* clade and with a basal split between the *K*. *puravida* lineage and a clade comprising *Kiwa* sp. GM and *K*. *araonae*. The weaker support for the sister-phyly of *Kiwa* sp. GM and *K*. *araonae* within *Bristly* Kiwaidae likely indicates phylogenetic incongruence across the different gene fragments, probably as a result of incomplete lineage sorting or hybrid introgression during the divergence of the three lineages. It may be, again, that the use of more gene loci, or even genomic data, may improve confidence in the topology, but if *Bristly* Kiwaidae diverged very rapidly yielding a short interval between nodes (see later discussion on divergence dates), this may not be the case.

The Pacific location of *Kiwa* sp. GM on the Galapagos Microplate, along with the location of *K*. *araonae* in the Pacific sector of the Southern Ocean on the Australian-Antarctic Ridge (AAR) tallies with previous interpretations [26,70], based on the Pacific locations of *K*. *puravida* and *K*. *hirsuta*, that Kiwaidae likely originated in the Pacific. This is also consistent with the assignment of the Alaskan fossil *Pristinaspina gelasina* on the stem of Kiwaidae [66]. Roterman *et al*. [26] further speculated, based on the location of *P*. *gelasina* and the basal split between the Costa Rican *K*. *puravida* and other kiwaids, that Kiwaidae may have originated in the Northern Hemisphere. However, given the large time interval between *P*. *gelasina* and the inferred age for the common ancestor of Kiwaidae, and a basal split between the Southern Hemisphere *Plumose* clade and a *Bristly* clade found in both hemispheres, we find the hypothesis of a Northern Hemisphere origin tenuous. Rather, the equatorial location of the two Northern Hemisphere species (*K*. *puravida* and *Kiwa* sp. GM), combined with the more southern distribution of all other species more likely indicates a Southern Hemisphere location for the common ancestor. Likewise, the East Pacific location of three of the four species in the Pacific (or Pacific sector of the Southern Ocean) points towards an East Pacific origin for Kiwaidae. A SE Pacific origin in the early-to-mid Cenozoic, chimes with palaeontological data indicating the importance of the Southern Hemisphere mid and high latitudes in the origin of many extant decapod genera from the Jurassic to the early Cenozoic [71].

**Fig 7:**
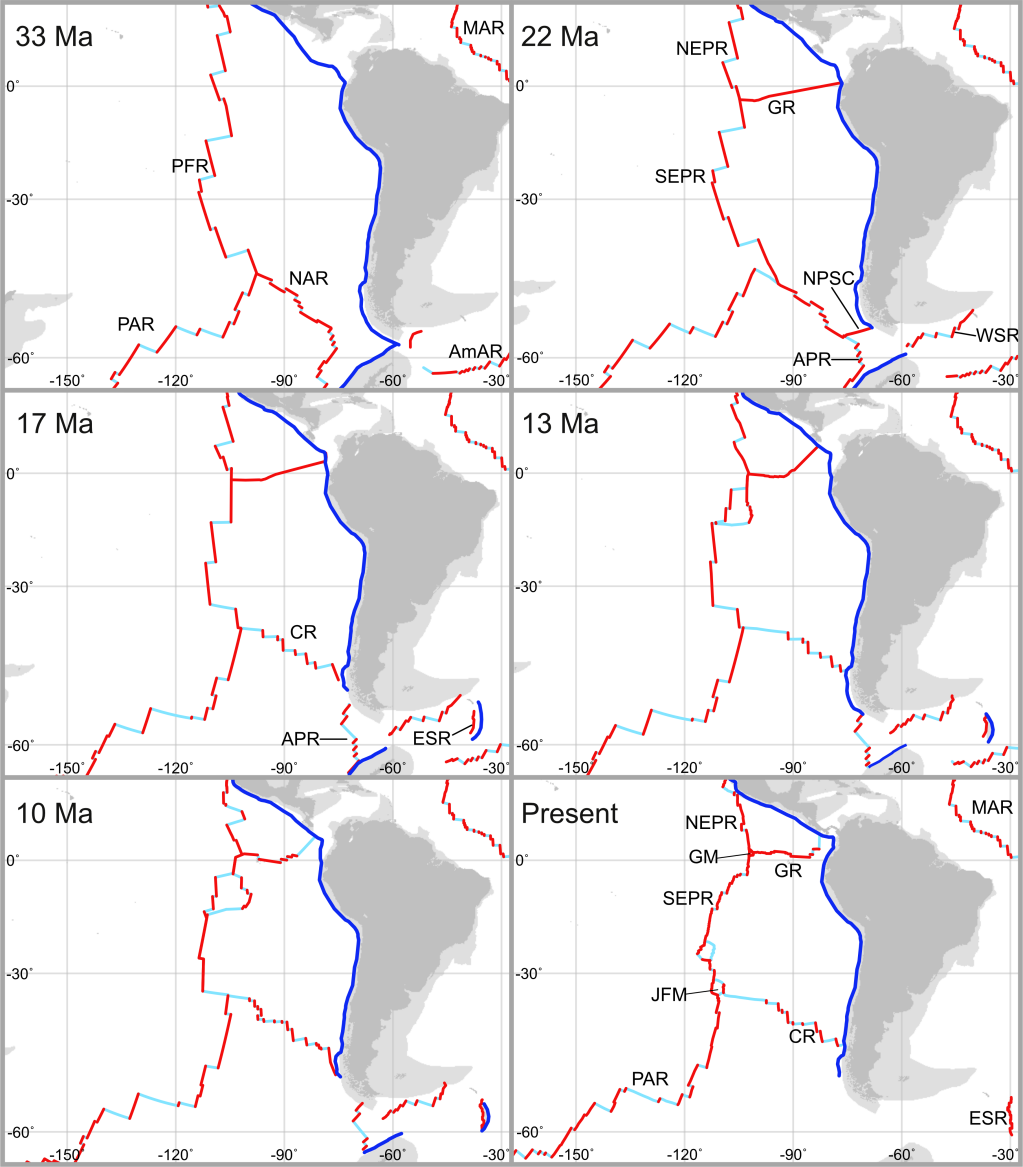
Schematic representing the evolution of ridge positions in the East Pacific and Southern Ocean relevant to the radiation of Kiwaidae in the Cenozoic. Taken from Matthews *et al*. [81] and Müller *et al*. [82] as rendered on the software GPlates 2.0 (https://www.gplates.org/index.html). Red lines denote active spreading segments of mid-ocean ridges and light blue lines represent faults and fracture zones. Darker Blue lines denote subduction zones. Light grey areas represent continental crust and dark grey areas show land masses with the present-day coastline. Abbreviations are: PFR = Pacific-Farallon Ridge; PAR = Pacific-Antarctic Ridge; NAR = Nazca-Antarctic Ridge; AmAR = American-Antarctic Ridge; NEPR = Northern East Pacific Rise, SEPR = Southern East Pacific Rise, GR = Galapagos Rift; NPSC = Nazca-Phoenix Spreading Centre; APR = Antarctic-Phoenix Ridge; WSR = West Scotia Ridge; CR = Chile Rise; ESR = East Scotia Ridge; MAR = Mid-Atlantic Ridge; JFM = Juan Fernandez Microplate; GM = Galapagos Microplate.

Roterman *et al*. [26] also argued, based on the basal split between a vent-associated clade and the seep-inhabiting *K*. *puravida*, that chemosynthetic Kiwaidae may have originated at hydrocarbon cold seeps and then transitioned to hydrothermal vents over evolutionary time; most likely along the East Pacific margin. However, the vent association of two of the three species within the *Bristly* clade would suggest either two independent transitions from seeps to vents within Kiwaidae, or more parsimoniously, that the common ancestor of extant Kiwaidae inhabited vents, with the *K*. *puravida* lineage subsequently transitioning from vents to seeps. Support for this model comes from the discrete trait reconstruction in the BEAST analysis producing a > 95% likelihood of “vent” association for the common ancestor of Kiwaidae, as opposed to “seep”, “coral” or “other” habitat types (S6 Table). The notion that deep-sea vent-endemic fauna may have derived from seep-associated taxa, which in turn derived from taxa associated with organic food fall and shallow-water habitats [72] is supported by early phylogenetic analyses with chemosynthetically-associated siboglinid polychaetes and mytilid mussels [12,13,16,73–75]. Recent studies with broader taxon sampling have tended to reveal more complicated patterns of habitat evolution, with vent-to-seep reversals and evidence of multiple radiations in mytilid mussels [14,15], and no clear pattern of habitat progression in vesicomyid clams [17], although a phylogenomic study of siboglinid polychaetes does indicate that vent-endemic species are the most derived clade, with the seep-endemic *Lamellibrachia* as basal [11], consistent with a seep-to-vent progression. Additionally a fossil squat lobster *Shinkaia katapsyxis* was found in Eocene seep deposits in Washington State, USA [76] and appears remarkably similar in form to the extant *Shinakaia crosnieri* which is found predominantly at vents East and South China Seas [77], although individuals have recently been discovered at seeps as well [78]. The low number of kiwaid species known to science presently, combined with the large time gap between *P*. *gelasina* from Cretaceous continental slope deposits and the putative Eocene-Oligocene age of the common ancestor of extant taxa (see later), leaves the question of how and where Kiwaidae first came to inhabit chemosynthetic ecosystems open for the time being. Whether the common ancestor of Kiwaidae inhabited seeps or vents (or both, or neither), the location of *K*. *puravida* on seeps close to where a formerly active spreading portion of the Galapagos Rift was being subducted under the Caribbean plate may be noteworthy (Figs 2 and 7). Network analysis indicates that regions where venting and seeps are in close proximity, e.g. where vents are close to subduction zones, are where habitat transitions are most likely to have occurred [79,80].

### Divergence comparisons

The key divergence date estimates produced in this study and those of Roterman *et al*. [26] are largely similar. In broad terms, the current analyses generated slightly older ages compared to the previous study. Both studies produced a Cretaceous origin for Chirostyloidea, for example, but instead of a median age of 114.8 Ma, these analyses resulted in older median ages ranging from 127.1–134.1 Ma. The MRCA of Kiwaidae was originally dated to 30.6 Ma in the early Oligocene, but the current study produced median estimates of 33.2–38.8 Ma, straddling the boundary between the Eocene and the Oligocene. Conversely, the current analyses produced both younger and older Miocene age estimates for the divergence of the *K*. *hirsuta* lineage and those of *K*. *tyleri* and *Kiwa* sp. SWIR (17.1–21.7 Ma), compared with the previous study (19.1 Ma). More importantly, whereas in the earlier analysis different chirostyloid tree topologies were not tested, in this study we are able to show that changing the sister family to Kiwaidae had only a minimal effect on the age estimates of kiwaid nodes, with differences ranging in the order of tens-of-thousands to hundreds-of-thousands of years at the most (S6 Table). The Cretaceous divergence estimates for the MRCA of Chirostyloidea produced here as well as by Roterman *et al*. [26] were also similar to that produced by Bracken-Grissom *et al*. [52] with a five-gene Anomura dataset (16S, 12S, H3, 18S and 28S) using 29 fossil calibrations. This similarity despite the use of some different markers and a more taxonomically broad fossil calibration scheme indicates that the internal kiwaid divergence estimates produced here are reasonably robust and unlikely to change substantially in the future, unless new fossils are discovered that radically push the age of calibrated nodes much further back in time.

### Kiwaid radiation

The Cenozoic radiation of Kiwaidae ~33–39 Ma (median estimates) chimes with evidence suggesting the majority of deep-sea chemosynthetic invertebrate groups radiated subsequent to the Palaeocene/Eocene Thermal Maximum (PETM) ~55 Ma; a warm climate episode that may have left much of the deep-sea floor suboxic or anoxic [9]. Such conditions may have pushed many deep-sea vent- and seep-endemic fauna–already persisting in narrow redox zones–beyond their physiological tolerances for low oxygen conditions, or at least reduced the availability of food through a drop in chemosynthetic primary productivity. The kiwaid radiation appears to have occurred ~20 million years after this event and represents one of the more recent post-PETM radiations for chemosynthetic fauna, along with bresiliid shrimp and bythograeid crabs, which may have radiated as recently as the Miocene ~10–20 Ma [9]. Coincident with the onset of the kiwaid radiation at the Eocene-Oligocene boundary is a sudden, substantial deepening of the carbonate compensation depth in the tropical East Pacific [83] from ~3.5 km to 4.5 km. This deepening likely signified a transition to cooler, more mixed deep water in the world’s oceans from previously warmer, more stratified conditions [84]. It could be that prior to this event, deep-sea chemosynthetic systems, especially those in the East Pacific where oxygen levels are lower than elsewhere [85] were still hostile to stem-lineage kiwaids (and possibly other decapod crustaceans). Decapods house gills enclosed in branchial chambers which are ventilated by the beating of the paddle-like scaphognathite under the dorsal carapace. Those that are chemosynthetically-associated have greater scaphognathite and gill surface area for gas exchange compared to their non-chemosynthetic relatives [86] and the branchial chambers of kiwaids are substantially larger than those of the apparently non-chemosynthetic stem lineage *P*. *gelasina* [66]. However, the housing of gills internally and the energetic costs of beating larger scaphognathites may place tighter constraints on the efficiency of gas exchange in hypoxic conditions, compared to species that can have external gas-exchange organs such as annelids [86]. This hypothesised evolutionary constraint may curb the ability of chemosynthetic decapods to adapt to more extreme hypoxia or delay the recolonisation of chemosynthetic habitats after PETM-like events in comparison with other taxa; offering one explanation for why decapods appear to be amongst the most recent additions to deep-sea chemosynthetic ecosystems.

### Ridge vicariance

Within Kiwaidae, the pattern of divergence may well be closely linked to the evolution and movement of mid-ocean spreading ridges supporting hydrothermal vent habitats. This is based on the fact that–with the exception of *K*. *puravida*–kiwaids have only been found at hydrothermal vents along with the BEAST analyses indicating the common ancestor of the clade most likely inhabited vents. The patterns of species and population diversity in other vent-associated taxa also appear consistent with vicariance related to the movement and evolution of active spreading ridges [87–92]. Additionally, morphological analyses of kiwaid larvae suggest that they are likely to be, at most, demersal drifters, with a high probability of retention along ridge axes [30]; consistent with a model of ridge-mediated dispersal in this clade. Nevertheless, the future discovery of kiwaids at non-vent or non-chemosynthetic habitats, or at vents in non-ridge settings such as on seamounts, may prompt a reassessment of the likely pathways in which Kiwaidae have diversified. If the common ancestor of Kiwaidae did inhabit vents, based on an East Pacific origin ~30–40 Ma, the candidate locations for their origin are likely to be the Pacific-Farallon Ridge (the ancestor of the EPR and Northeast Pacific Ridges), the predecessor to the Chile Rise–the Nazca-Antarctic Ridge, or the Pacific-Antarctic Ridge (PAR) (Fig 7).

The primary divergence within the *Plumose* Kiwaidae clade, between the *K*. *hirsuta* lineage and the lineage comprising *K*. *tyleri* from the East Scotia Ridge (ESR) and *Kiwa* sp. SWIR appears to have occurred 17.1–21.7 Ma (median estimates), in the early-to-mid Miocene. Given the Southern hemisphere distribution of these species along with the Pacific locations of *K*. *hirsuta* and the *Bristly* clade, the most likely origin for the common ancestor of this group is at vents in the SE Pacific—either on the PAR, the southern EPR, the Chile Rise (and the former NAR) or on the smaller Antarctic-Phoenix Ridge (APR) or the Nazca-Phoenix Spreading Centre (NPSC) (Fig 7). With a similar MRCA age estimate to those presented here (19.1 Ma), Roterman *et al*. [26] proposed–based on the basal split between *K*. *hirsuta* and the other two species–that kiwaids moved from vents in the Pacific into the Atlantic sector of the Southern Ocean (and beyond) via now-extinct spreading ridges that bisected the Drake Passage, which itself was open by ~30 Ma [93] (Fig 7). The timing of the divergence between *K*. *hirsuta* and the other sister lineage is synchronous with the isolation of vents in the Drake Passage and Scotia Sea around 15–20 Ma [26] (Fig 7) and the divergence estimates generated herein remain consistent with this model. The median estimates for the divergence of *K*. *tyleri* and *Kiwa* sp. SWIR of 1.5–2.1 Ma (95% HPD range of 0.7–4.3 Ma) are again similar to that of Roterman *et al*. [26] (1.5 Ma), who observed that no obvious changes in ridge configuration between the ESR, the American-Antarctic Ridge (AmAR) and the SWIR could obviously account for this divergence through vicariance (Fig 2). They suggested instead that range-expansion and subsequent divergence could have been initiated either by changes in hydrothermal activity along intervening ridges, or changes in the latitude and intensity of the Antarctic Circumpolar Current during the Plio-Pleistocene, which might have strongly influenced the distance that larvae could be transported. Further exploration of the intervening ridges (American-Antarctic Ridge and southern parts of the SWIR) will provide clues as to the likely cause.

### *Bristly* radiation

At a similar time during the Miocene the *Bristly* group also appears to have radiated, with a primary split into two lineages 13.3–15.9 Ma (median estimate) and a second split shortly afterwards 10.1–13.5 Ma (HPD ranges of 7–21.8 Ma and 4.8–18.3 Ma respectively). The tree topology in the phylogenetic analyses offered some limited support for a primary split between the *K*. *puravida* lineage and another lineage subsequently splitting into the *Kiwa* sp. GM and *K*. *araonae* lineages, although we treat this splitting scheme with caution given their near simultaneous timing. Weak support for the tree topology presented here is to be expected if a rapid radiation has occurred, owing to incomplete lineage sorting of gene loci and the possibility of hybrid introgression after the process of divergence had begun. The swift radiation of *Bristly* Kiwaidae coincides with another large global shift in oceanographic conditions during the middle Miocene ~12–16 Ma, known as the middle Miocene Climate Transition (MMCT) [94]. This episode was characterised by a substantial cooling and increased ventilation of the world’s deep-water masses and a rearrangement of global ocean currents [95] following a 2 My warm episode that had resulted in a ~600 m shoaling of the carbonate compensation depth in the tropical East Pacific [83,96]. If the tree topology presented here is taken at face value, the most probable location for the primary split within the group is near the Equator in the East Pacific (Figs 2 and 7), with a subsequent southward migration for the *K*. *araonae* lineage, although we acknowledge that other scenarios could exist, especially with a different sequence of divergence events in the clade. For example, a basal split between the *K*. *araonae* lineage and a clade comprising *Kiwa* sp. GM and *K*. *puravida* would make a southern location for the initial split more plausible. Regardless of the precise sequence of divergence, the timing of the divergence of *K*. *puravida* from other vent-associated *Bristly* kiwaids 4.8–21.8 Ma (combined HPD estimates), coincides with changes that occurred on the Galapagos Rift during the Miocene epoch. This spreading ridge formed ~23 Ma and once stretched from the Galapagos Triple Junction–near the present-day location of *Kiwa* sp. GM–all the way to the subducting Caribbean plate margin–near the present-day location of *K*. *puravida* [97,98]. Around 14.5–14.7 Ma there was a change in the ridge spreading axis from SW-NE to roughly W-E and the appearance of fracture zones offsetting ridge segments, which could have resulted in disrupted larval connectivity across the ridges, leading to divergence between the seep and vent *Bristly* lineages. Alternatively, the appearance of the Panama fracture zone around by ~10 Ma heralded the extinction of the ridge segment closest to the shelf, resulting in a ~700 km gap between the continental shelf and the nearest active spreading ridge [97] (Fig 7). Whether or not any of these scenarios are in fact the case, as with the initial radiation of the Kiwaidae before, the onset of the *Bristly* radiation during a cooling transition from a warmer episode could be significant. Large changes in oceanographic conditions might have spurred the demographic expansion and subsequent diversification of kiwaids in the East Pacific and it is noteworthy that the middle Miocene also appears to have been an important period in the geographical spread and diversification of clades and sub-clades within hydrothermal vent barnacles and vesicomyid clams [17,87].

### Presence and absence

**Fig 8:**
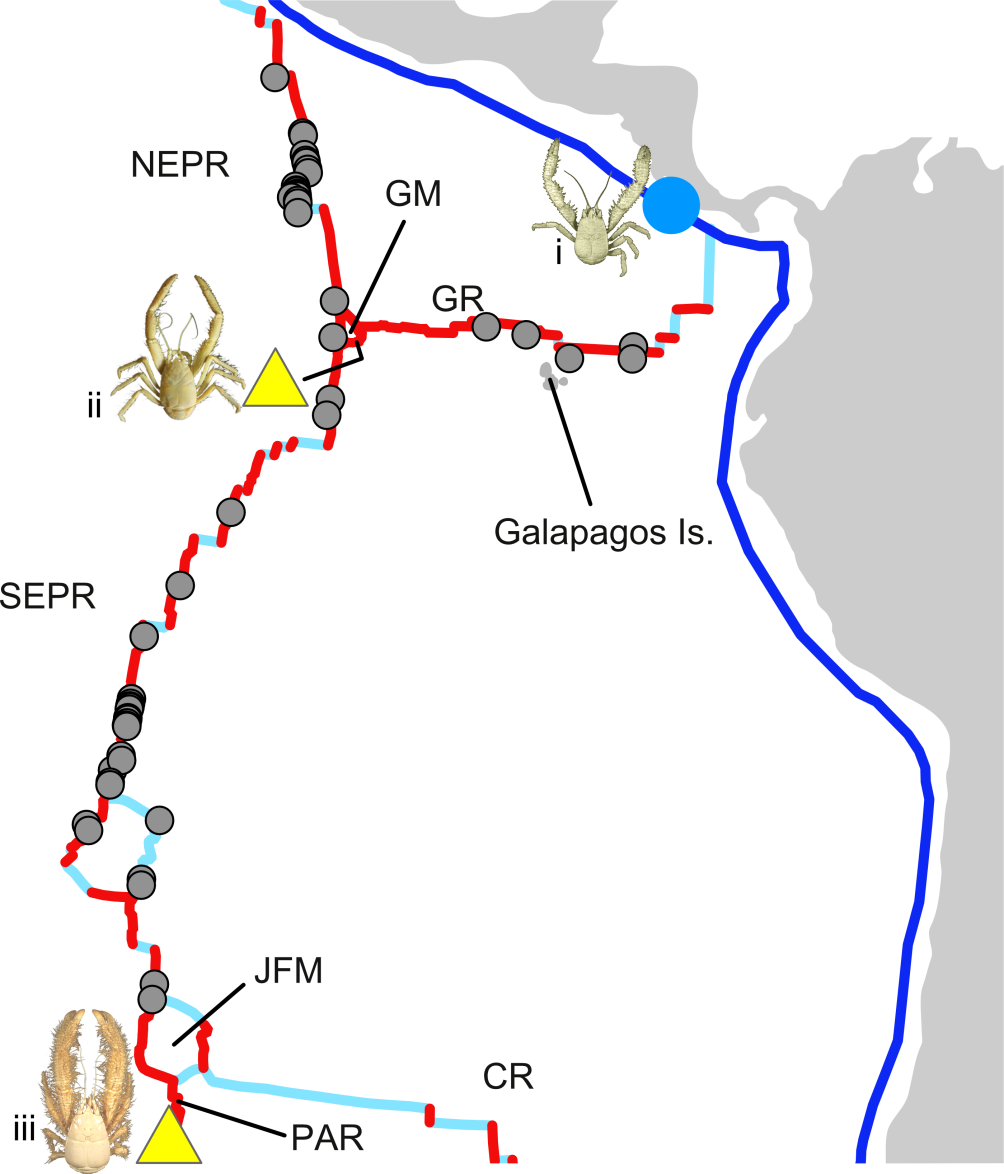
Schematic showing the present-day configuration of mid-ocean ridges in the tropical East Pacific and the location of known kiwaids. Modified from Matthews *et al*. [81] and Müller *et al*. [82] as rendered on the software GPlates 2.0 (https://www.gplates.org/index.html). Grey spots represent hydrothermal vents that have been visually surveyed, according to the InterRidge Vents Database version 2.1 [99], but where no kiwaids have been found. Yellow triangles and blue circles show hydrothermal vents and hydrocarbon seeps (respectively) where kiwaids have been found. i = *Kiwa puravida*; ii = *Kiwa* sp. GM; iii = *Kiwa hirsuta*. Grey areas represent land. Red lines denote active spreading segments of the ridges. Light blue lines represent faults and fracture zones. Darker Blue lines denote subduction zones. Abbreviations are: NEPR = Northern East Pacific Rise; SEPR = Southern East Pacific Rise; GR = Galapagos Rift; JFM = Juan Fernandez Microplate; GM = Galapagos Microplate; PAR = Pacific-Antarctic Ridge; CR = Chile Rise. Photograph of *K*. *puravida* modified from Thurber *et al*. [19] (CC BY 4.0) and *Kiwa hirsuta* modified from Muséum National D’Histoire Naturelle (MNHN) crustacean collection–credit Noémy Mollaret (CC BY 4.0).

The present-day locations of Kiwaidae are, to some extent puzzling. Whilst there are vast regions of the World’s oceans yet to be explored where kiwaids may be found (Fig 2), there are also regions where kiwaids are conspicuously absent where they would otherwise be expected. Although *K*. *puravida* and *Kiwa* sp. GM are located adjacent to the Galapagos Rift, and *Kiwa* sp. GM and *Kiwa hirsuta* adjacent to the southern EPR, one might expect to find kiwaids at vents along both the Galapagos Rift and the southern EPR, but despite vents in these two regions being extensively explored [72,89], no kiwaids have been found so far (Fig 8). If vent-associated kiwaids have primarily spread via mid-ocean ridges, then the present distribution of kiwaids indicates that they are likely to have previously existed along portions of mid-ocean ridge in the tropical East Pacific where they are currently absent. The presence of *K*. *hirsuta*, part of the *Plumose* clade on the PAR, in close proximity to the Chile Rise, with *Bristly* kiwaids either side of it (albeit far away) on the AAR and Galapagos Microplate (*Kiwa araonae* and *Kiwa* sp. GM respectively–Fig 2) is another hint that the species distributions have shifted in the past. Further exploration of the PAR and the Chile Rise revealing the current distribution of the *Bristly* and *Plumose* clades may yield clues as to where they initially diverged and how they came to be in their current locations. Exploration northeast of *K*. *araonae* on the AAR and also on the Southeast Indian Ridge should reveal both the easterly and westerly extent of *Bristly* kiwaids.

### Extinction, recolonisation and radiation

Historic regional extinctions and recolonisations have been proposed to account for the patterns of genetic diversity amongst subpopulations of *Bathymodiolus* mussels and *Tevnia jerichonana* tubeworms between the PAR and the northern EPR [100,101]. This pattern is best explained by repeated extinctions along the intervening southern EPR, followed by recolonisation and hybridisation between southern and northern populations. The cause of these extinctions has been attributed to the high frequency of eruptions, and presumed shorter vent field longevity along the southern EPR [100–102], which exhibits the fastest spreading rates on the planet (~150 mm yr^-1^) [103]. The factors that determine the viability of metapopulations along a stretch of mid-ocean ridge are myriad: e.g. life history and dispersal traits, sea bottom currents, topographical barriers to dispersal, and the ephemerality and the density of the vent fields along the ridge, amongst others [89]. Modelling has shown that, given a particular mode of dispersal, the persistence of a vent metapopulation may be highly dependent on the rate and spatial pattern of vent field birth and death; with metapopulation connectivity mediated via “transient contact zones” between shifting habitat ranges over time [104]. *Kiwa tyleri* larvae, which are lecithotrophic and likely demersally drifting, exhibit extremely abbreviated development; hatching as megalopa following an extensive (> 1 year) brooding period [30,31]. This mode of dispersal seems optimised for local retention on patchy habitats, while not entirely excluding the possibility of occasional longer-range dispersal. Such a mode of dispersal could render a metapopulation particularly vulnerable to stochastic fragmentation and regional extinction on a fast-spreading ridge where eruptions may periodically resurface large areas; potentially accounting for the absence of kiwaids on the EPR. The appearance of further barriers to dispersal, such as microplates and fracture zones could hinder subsequent recolonisation after a regional extinction and it is noteworthy that *K*. *hirsuta* is not found north of the Juan Fernandez Microplate, which formed ~4 Ma [105] and that *Kiwa* sp. GM is itself apparently isolated on the Galapagos Microplate which formed ~1.5 Ma [106].

Low temperatures have been shown to extend longevity in vent polychaete larvae [107] and in Antarctic echinoderms [108] through the reduction of metabolic activity (and hence food and oxygen requirements) and it has been suggested that cold bottom temperatures in the Scotia Sea (< 0˚C) may therefore facilitate connectivity between *Kiwa tyleri* colonies across the East Scotia Ridge [23,31,32]. Conversely, the warmer bottom waters in the tropical East Pacific could further limit the dispersal capability of kiwaid larvae and lower ambient oxygen levels and higher bottom water temperatures in the past could have exacerbated this. Whilst a limited dispersal capability might leave kiwaids populations more vulnerable to fragmentation on geologically volatile stretches of ridge, it could allow population fragments isolated on more stable “oases” of chemosynthetic activity to persist through local recruitment. The half spreading rates at the eastern end of the Galapagos Rift and on the Galapagos Microplate (~26–48 mm yr^-1^) are a fraction of other rates in the region [103] and spreading rates on the northern PAR (94 mm yr^−1^) are also substantially slower than the SEPR, which could be indicative of greater vent field longevity. Seeps are also generally considered more temporally stable than vents [3]. Greater habitat longevity at these sites could explain how *Kiwa* sp. GM and *K*. *puravida* appear to have persisted in small patches and how *Kiwa hirsuta* exists south of the Juan Fernandez Microplate. The size of vent-associated kiwaid metapopulations and the continuity of their species ranges in unexplored regions of mid-ocean ridge may therefore largely reflect the interplay between ambient seafloor temperatures, the degree of deep-water ventilation, and ridge spreading rate (as a proxy for vent field ephemerality).

More generally then, the patchy distribution of Kiwaidae in the tropical East Pacific could be a snapshot of the stochastic ebbing and flowing of ranges along fast-spreading ridges that characterises other vent-endemic species in the region to differing degrees and periodicities as a consequence of intrinsic life history differences. This ebb and flow could be further punctuated by large-scale climate fluctuations, or by changes in ridge spreading rates, as has occurred along the Pacific-Farallon ridge during the Cenozoic [109]. The current distribution, pattern of diversity, tree topology and divergence timing of Kiwaidae, therefore, appears consistent with cycles of extinction, range expansion and lineage divergence along large segments of mid-ocean ridge. The exploration of the unknown portions of mid-ocean ridge, continental shelf margins and seamounts may yet yield surprises that will necessitate a substantial revision of the evolutionary history of kiwaids. However, if subsequent discoveries do not radically change our present expectations regarding the distribution of kiwaids around the globe, then present-day kiwaid biogeography may exemplify the transience of species ranges on mid-ocean ridge hydrothermal vent systems, as well as the vulnerability of chemosynthetic metapopulations to large-scale regional extinction.

## Conclusion

This study is an augmentation of Roterman *et al*. [26] through the addition of more kiwaids, longer alignments and more fossil calibrations. Tree topologies produced here modify some of the inferences of the previous study. The sister-phyly of Kiwaidae and Chirostylidae within Chirostyloidea is placed in doubt, as are the previous inferences of a seep-to-vent evolutionary progression and a Northern Hemisphere origin for Kiwaidae. Current analyses do support the earlier inference for an East Pacific origin, however, and divergence estimates are broadly similar to previous analyses. Age estimates for the MRCA of Kiwaidae indicate an origin long after the PETM, around the Eocene-Oligocene boundary at a time of deep-water cooling and increased ventilation in the Pacific. Likewise, the rapid radiation of a newly defined *Bristly* clade appears synchronous with another transition to cooler and more ventilated conditions in the East Pacific during the Middle Miocene. The distribution, diversity, tree topology and divergence timing of vent-associated Kiwaidae in the Pacific is consistent with a pattern of regional extinctions, recolonisations and radiations along fast-spreading ridges over the last 40 million years. This pattern may have been punctuated by large-scale fluctuations in deep-water ventilation and temperature during the Cenozoic; further affecting the viability of Kiwaidae populations along large areas of mid-ocean ridge. The exploration of new vent and seep systems in the Pacific and beyond will help to better resolve the biogeographic history of Kiwaidae and provide new insights into the long-term resilience of metapopulations inhabiting deep-sea chemosynthetic ecosystems.

## Supporting information

S1 File Supplementary materials and methods. (DOCX)

S1 Table Classification, sampling locations/provenance and voucher ID of the species and GenBank accession numbers of genes used in this study. 'X' denotes missing data. GenBank accession numbers in bold are new sequences from this study and italicised numbers are existing genbank sequences that have been extended.
(DOCX)

S2 Table List of primers used in this study. (DOCX)

S3 Table Gblocks 0.91 scheme for excising poorly or ambiguously aligned portions of rRNA sequences. (DOCX)

S4 Table Substitution models used in this study as determined by PartitionFinder 2.1.1. (DOCX)

S5 Table Predominant habitats as reported in publications–or observed during specimen collection—along with habitat designation in Beast 2.4.3 discrete trait analyses. (DOCX)

S6 Table Output from Beast 2.4.3 analyses showing divergence date estimates and % probability of habitat assignment for key nodes within Chirostyloidea and Kiwaidae. (DOCX)

S1 Fig Maximum likelihood tree topology (best ML tree) of a nine-partition anomuran crustacean dataset generated in IQ-TREE 1.5.4. Node support numbers represent ML bootstrap percentages from 1000 non-parametric bootstrap replicates.
(TIFF)

S2 Fig Maximum likelihood tree topology (best ML tree) of a fourteen-partition anomuran crustacean dataset generated in IQ-TREE 1.5.4. Node support numbers represent ML bootstrap percentages from 1000 non-parametric bootstrap replicates.
(TIFF)

S3 Fig Maximum likelihood tree topology with the ultrafast bootstrap approximation method of a nine-partition anomuran crustacean dataset generated in IQ-TREE 1.5.4. Node support numbers represent ultrafast approximate bootstrap percentages from 100,000 replicates.
(TIFF)

S4 Fig Maximum likelihood tree topology with the ultrafast bootstrap approximation method of a fourteen-partition anomuran crustacean dataset generated in IQ-TREE 1.5.4. Node support numbers represent ultrafast approximate bootstrap percentages from 100,000 replicates.
(TIFF)
