# Supplementary material for: A new yeti crab phylogeny: Vent origins with indications of regional extinction in the East Pacific
Source: PLoS One. 2018 Mar 16;13(3):e0194696. doi: 10.1371/journal.pone.0194696 (PMC5856415; doi:10.1371/journal.pone.0194696)
Supplement: S1 File — (DOCX) [file pone.0194696.s001.docx]

# Supplementary Materials & Methods

For *Kiwa araonae* tissue, PCR reactions were carried out in 20 µl volumes containing 2 µl of template DNA, 2 µl of 10x nTaq-Tenuto buffer (Mg²+plus), 1 µl of dNTP mix at a concentration of 2 mM/µl, 1 µl of primers at a concentration of 10 pmol/µl, 1 µl of 1xBSA (bovine serum albumin), 0.6 unit of nTaq-Tenuto DNA polymerase (Enzynomics, Daejeon, Korea) and 11.88 µl of double distilled water). General amplification conditions were initial denaturation at 94 °C for 15 minutes, followed by 35 cycles of a denaturation step at 94 °C for 1 minute, an annealing step at 55-60 °C depending on the gene (see table below) for 1.5 minute and an elongation step at 72 °C for 1 minute. The final elongation step was at 72 °C for 10 minutes and the samples were stored at 4 °C until displaced. All reactions were performed on a Bio-Rad Peltier Thermal Cycler 100. Purification of PCR products were carried out using LaboPass™ PCR Purification Kit (Cosmogenetech, Seoul, Korea) following the manufacturer’s instructions. DNA sequencing was carried out with an Applied Biosystems 3730xl automatic sequencer.

PCR cloning was performed for nuclear genes, Enol, PEPCK, and AK, where ambiguous chromatogram peaks were indicative of single nucleotide polymorphisms within heterozygote individuals. Cloning was conducted using TOPcloner TA kit (Enzynomics, Daejoen, Korea). Initially, 0.5-4 µl of PCR product was mixed with 1 µl of 6X TOPcloner buffer, 1 µl of pTOP TA V2 vector, and distilled water (making up a total of 6 µl) and incubated at 37 °C for 5 minutes. Then, the mixture was added to 100 µl of competent *Escherichia coli* cells for transformation. The transformed cells were plated on Luria-Bertani (LB) agar plates with 100 µg/mL ampicillin. This was grown overnight (12-16 hours) at incubator (37 °C). Five to ten positive colonies were picked for each gene and added to FastMix/Frenche ™ PCR Kit (iNtRON Biotechnology, Seongnam, Republic of Korea) with 1 µl of primers M13F (-20) and M13R (-40) and 15 µl of distilled water. The PCR conditions were initial denaturation at 94 °C for 10 minutes, followed by 35 cycles of a denaturation step at 94 °C for 30 seconds, an annealing step at 50 °C for 30 seconds, and an elongation step at 72 °C for 1 minute. The final elongation step was at 72 °C for 10 minutes. The two PCR products with the clearest bands shown with gel electrophoresis on agarose gel extracted using QIAquick Gell Extraction Kit (Qiagen, Hilden, Germany) following manufacturer’s direction. Sequencing was carried out by Applied Biosystems 3730xl automatic sequencer.

For *Kiwa* sp. GM, PCR amplifications were carried out using 1×PCR buffer, 50 µM dNTP mixture, 0.2 μM of each primer, 10 ng of template genomic DNA, and 1 U of ExTaq DNA Polymerase (Takara, Tokyo, Japan) in 50 μL reactions. The thermal cycle procedure was 94ºC for 60 seconds, followed by 30 cycles of 94ºC for 20 seconds, 50ºC for 30 seconds, 72ºC for 90 seconds, and a final extension of 2 min at 72ºC with a Mastercycler (Eppendorf, Hamburg, Germany). The annealing temperature was 52°C for 16S. All PCR product was purified using a DNA purification kit (Shangong, Shanghai, China) and sequenced directly in both directions on an ABI PRISM 3730XL (Applied Biosystems, Foster City, CA, USA) following the manufacturer’s instructions. For other tissue, PCR and sequencing conditions were performed as per Roterman *et al.* (2013).

## References

Roterman CN, Copley JT, Linse KT, Tyler PA, Rogers AD (2013) The biogeography of the yeti crabs (Kiwaidae) with notes on the phylogeny of the Chirostyloidea (Decapoda: Anomura). Proceedings. Biological sciences / The Royal Society, 280, 20130718–20130718.
