# Supplementary material for: A new yeti crab phylogeny: Vent origins with indications of regional extinction in the East Pacific
Source: PLoS One. 2018 Mar 16;13(3):e0194696. doi: 10.1371/journal.pone.0194696 (PMC5856415; doi:10.1371/journal.pone.0194696)
Supplement: S1 Table — 'X' denotes missing data. GenBank accession numbers in bold are new sequences from this study and italicised numbers are existing genbank sequences that have been extended. (DOCX) [file pone.0194696.s002.docx]

Table S1. Classification, sampling locations or provenance and voucher ID of the species and GenBank accession numbers of genes used in this study. 'X' denotes missing data. GenBank accession numbers in bold are new sequences from this study and italicised numbers are existing genbank sequences that have been extended.
