# Supplementary material for: A new yeti crab phylogeny: Vent origins with indications of regional extinction in the East Pacific
Source: PLoS One. 2018 Mar 16;13(3):e0194696. doi: 10.1371/journal.pone.0194696 (PMC5856415; doi:10.1371/journal.pone.0194696)
Supplement: S2 Table — (DOCX) [file pone.0194696.s003.docx]

Table S2. List of Primers used in this study.

Ahyong, S. T. and D. O'Meally (2004). Phylogeny of the decapoda reptantia: Resolution using three molecular loci and morphology. Raffles Bulletin of Zoology 52(2): 673-693.

Ahyong, S.T., Schnabel, K.E. & Maas, E. (2009) Anomuran phylogeny: new insights from molecular data. In: Martin, J.W., Crandall, K.A. & Felder, D.L. (eds.), Decapod Crustacean Phylogenetics. CRC Press, Boca Raton, Florida, pp. 399-414.

Folmer O, Black M, Hueh W, Lutz R, Vrijenhoek R (1994) DNA primers for amplification of mitochondrial cytochrome c oxidase subunit I from diverse metazoan invertebrates. Mol Mar Biol Biotechnol 3: 294-299.

Halanych, K. M., R. A. Lutz and R. C. Vrijenhoek (1998). Evolutionary origins and age of vestimentiferan tube-worms. Cahiers De Biologie Marine 39(3-4): 355-358.

Rogers AD, Tyler PA, Connelly DP et al. (2012) The discovery of new deep-sea hydrothermal vent communities in the Southern Ocean and implications for biogeography. Plos Biology, 10, e1001234.

Roterman CN, Copley JT, Linse KT, Tyler PA, Rogers AD (2013) The biogeography of the yeti crabs (Kiwaidae) with notes on the phylogeny of the Chirostyloidea (Decapoda: Anomura). Proceedings. Biological sciences / The Royal Society, 280, 20130718–20130718.

Tsang LM, Ma KY, Ahyong ST, Chan TY, Chu KH (2008) Phylogeny of Decapoda using two nuclear protein-coding genes: Origin and evolution of the Reptantia. Molecular Phylogenetics and Evolution, 48, 359–368.

Tsang, L. M., T-Y. Chan, S. T. Ahyong and K. H. Chu (2011). Hermit to king, or hermit to all: Multiple transitions to crab-like forms from hermit crab ancestors. Systematic Biology 60(5): 616-629.
