# Supplementary material for: A new yeti crab phylogeny: Vent origins with indications of regional extinction in the East Pacific
Source: PLoS One. 2018 Mar 16;13(3):e0194696. doi: 10.1371/journal.pone.0194696 (PMC5856415; doi:10.1371/journal.pone.0194696)
Supplement: S3 Table — (DOCX) [file pone.0194696.s004.docx]

Table S3. Gblocks 0.91 scheme for excising poorly or ambiguously aligned portions of rRNA sequences.
