# Supplementary material for: A new yeti crab phylogeny: Vent origins with indications of regional extinction in the East Pacific
Source: PLoS One. 2018 Mar 16;13(3):e0194696. doi: 10.1371/journal.pone.0194696 (PMC5856415; doi:10.1371/journal.pone.0194696)
Supplement: S5 Table — (DOCX) [file pone.0194696.s006.docx]

Table S5. Predominant habitats as reported in publications or observed during specimen collection along with the habitat designation in Beast 2.4.3 discrete trait analyses.

Ardika PU, Farajallah A, Wardiatno Y (2015) First Record of Hippa adactyla (Fabricius, 1787; Crustacea, Anomura, Hippidae) from Indonesian Waters. Tropical Life Sciences Research, 26, 105–110.

Baba K, Macpherson E, Lin CW, Chan TY (2009) Crustacean Fauna of Taiwan. Squat lobsters (Chirostylidae and Galatheidae). National Taiwan Ocean University, Taiwan.

Buhl-Mortensen L, Mortensen PB (2010) Crustaceans associated with the deep-water gorgonian corals Paragorgia arborea (L., 1758) and Primnoa resedaeformis (Gunn., 1763). Journal of Natural History, 38, 1233–1247.

Cabezas, P., Macpherson, E., & Machordom, A. (2008). A new genus of squat lobster (Decapoda: Anomura: Galatheidae) from the South West Pacific and Indian Ocean inferred from morphological and molecular evidence. Journal of Crustacean Biology, 28(1), 68-75.

Cabezas P, Sanmartín I, Paulay G, Macpherson E, Machordom A (2012) Deep under the sea: unraveling the evolutionary history of the deep‐sea squat lobster Paramunida (Decapoda, Munididae). Evolution, 66, 1878–1896.

Cartes JE, Papiol V, Frutos I et al. (2014) Distribution and biogeographic trends of decapod assemblages from Galicia Bank (NE Atlantic) at depths between 700 and 1800m, with connexions to regional water masses. Deep Sea Research Part II: Topical Studies in Oceanography, 106, 165–178.

Copley JT, Marsh L, Glover AG et al. (2016) Ecology and biogeography of megafauna and macrofauna at the first known deep-sea hydrothermal vents on the ultraslow-spreading Southwest Indian Ridge. Scientific Reports, 6, srep39158.

Davie PJ (2002) Zoological Catalogue of Australia-Malacostraca-Eucarida-Decapoda-Anomura, Brachyura (A Wells, W Houston, Eds,). CSIRO Publishing, Collingwood, Victoria, Australia.

Forest J (1987) Ethology and Distribution of Pylochelidae (Crustacea Decapoda Coenobitoidea). Bulletin of Marine Science, 41, 309–321.

Le Guilloux E, Hall-Spencer JM, Söffker MK, Olu K (2010) Association between the squat lobster Gastroptychus formosus and cold-water corals in the North Atlantic. Journal of the Marine Biological Association of the United Kingdom, 90, 1363–1369.

Haig J (1974) A review of the Australian crabs of family Hippidae (Crustacea, Decapoda, Anomura). Memoirs of the Queensland Museum.

Jara CG, López MT (1981) A new species of freshwater crab (Crustacea: Anomura: Aeglidae) from insular south Chile. Proceedings of the Biological Society of Washington, 94, 88–93.

Jonsson LG, Nilsson PG, Floruta F, Lundalv T (2004) Distributional patterns of macro- and megafauna associated with a reef of the cold-water coral Lophelia pertusa on the Swedish west coast. Marine Ecology-Progress Series, 284, 163–171.

Lancaster I (1988) Pagurus bernhardus (L.)—an introduction to the natural history of hermit crabs. Field Studies, 7, 189–238.

Lee S-H, Lee W-K, Won Y-J (2016) A new species of yeti crab, genus Kiwa Macpherson, Jones and Segonzac, 2005 (Decapoda: Anomura: Kiwaidae), from a hydrothermal vent on the Australian-Antarctic Ridge. Journal of Crustacean Biology, 36, 238–247.

Li HY, Lin FJ, Chan BKK, Chan TY (2008) Burrow morphology and dynamics of mudshrimp in Asian soft shores. Journal of Zoology, 274, 301–311.

Macpherson E, Baba K (1993) Crustacea Decapoda: Munida japonica Stimpson, 1858, and related species (Galatheidae). Mémoires du Muséum national d'histoire naturelle, 156, 381–420.

Macpherson E, Jones W, Segonzac M (2005) A new squat lobster family of Galatheoidea (Crustacea, Decapoda, Anomura) from the hydrothermal vents of the Pacific-Antarctic Ridge. Zoosystema, 27, 709–723.

Millikin MR, Williams, AB (1984) Synopsis of biological data on the blue crab, Callinectes sapidus Rathbun. NOAA Technical Report NMFS.

Minh BQ, Nguyen MAT, Haeseler von A (2013) Ultrafast Approximation for Phylogenetic Bootstrap. *Molecular Biology and Evolution*, **30**, 1188–1195.

Nicol EAT (1932) The Feeding Habits of the Galatheidea. Journal of the Marine Biological Association of the United Kingdom, 18, 87–106.

Ngoc-Ho N (2001) Austinogebia, a new genus in the Upogebiidae and rediagnosis of its close relative, Gebiacantha Ngoc-Ho, 1989 (Crustacea: Decapoda: Thalassinidea). In: Advances in Decapod Crustacean Research, pp. 47–58. Springer Netherlands, Dordrecht.

Osawa M (2007) A new species of Chirostylus Ortmann, 1892 (Crustacea: Decapoda: Anomura: Chirostylidae) from the Ryukyu Islands, southwestern Japan, with a supplemental description of Chirostylus ortmanni Miyake & Baba, 1968. Zootaxa, 1450, 31–43.

Oyanedel A, Valdovinos C, Sandoval N et al. (2011) The Southernmost Freshwater Anomurans of the World: Geographic Distribution and New Records of Patagonian Aeglids (Decapoda: Aeglidae). Journal of Crustacean Biology, 31, 396–400.

Panning A (1938) The Chinese mitten crab. Smithsonian Rep, 361–375.

Poupin J, Lemaitre R (2014) Porcellanid crabs from Guadeloupe Island (Crustacea, Decapoda, Anomura), with an updated list of species from the Lesser Antilles. dx.doi.org, 36, 5–27.

Prakash S, Kumar TTA, Khan SA (2013) Checklist of the Porcellanidae (Crustacea: Decapoda: Anomura) of India. Check List, 9, 1514–1518.

Rogers AD, Tyler PA, Connelly DP et al. (2012) The discovery of new deep-sea hydrothermal vent communities in the Southern Ocean and implications for biogeography. Plos Biology, 10, e1001234.

Roterman CN, Copley JT, Linse KT, Tyler PA, Rogers AD (2013) The biogeography of the yeti crabs (Kiwaidae) with notes on the phylogeny of the Chirostyloidea (Decapoda: Anomura). Proceedings. Biological sciences / The Royal Society, 280, 20130718–20130718.

Shima T, Kakinuma Y (1993) Symbiotic life of porcelain crab Neopetrolisthes maculatus and the sea anemone Stichodactyla gigantea. Zoological Science, 10, 164.

Telford M, Daxboeck C (1978) Porcellana sayana Leach (Crustacea: Anomura) symbiotic with Strombus gigas (Linnaeus)(Gastropoda: Strombidae) and with three species of hermit crabs (Anomura: Diogenidae) in Barbados. Bulletin of Marine Science, 28, 202–205.

Thurber AR, Jones WJ, Schnabel K (2011) Dancing for food in the deep sea: bacterial farming by a new species of yeti crab. Plos One, 6, e26243.

Veilleux É, De Lafontaine Y (2007) Biological synopsis of the Chinese mitten crab (Eriocheir sinensis). Fisheries and Oceans Canada, Nanaimo, British Columbia.

Wang J, Lin R, Bamber RN, Huang D (2013) Two new species of Sericosura Fry & Hedgpeth, 1969 (Arthropoda: Pycnogonida: Ammotheidae) from a hydrothermal vent on the East Pacific Rise. Zootaxa, 3669, 165–171.

Yang C-H, Tsuchida S, Fujikura K et al. (2016) Connectivity of the squat lobsters Shinkaia crosnieri (Crustacea: Decapoda: Galatheidae) between cold seep and hydrothermal vent habitats. Bulletin of Marine Science, 92, 17–31.
