# Supplementary figures and images for: A new yeti crab phylogeny: Vent origins with indications of regional extinction in the East Pacific
Source: PLoS One. 2018 Mar 16;13(3):e0194696. doi: 10.1371/journal.pone.0194696 (PMC5856415; doi:10.1371/journal.pone.0194696)

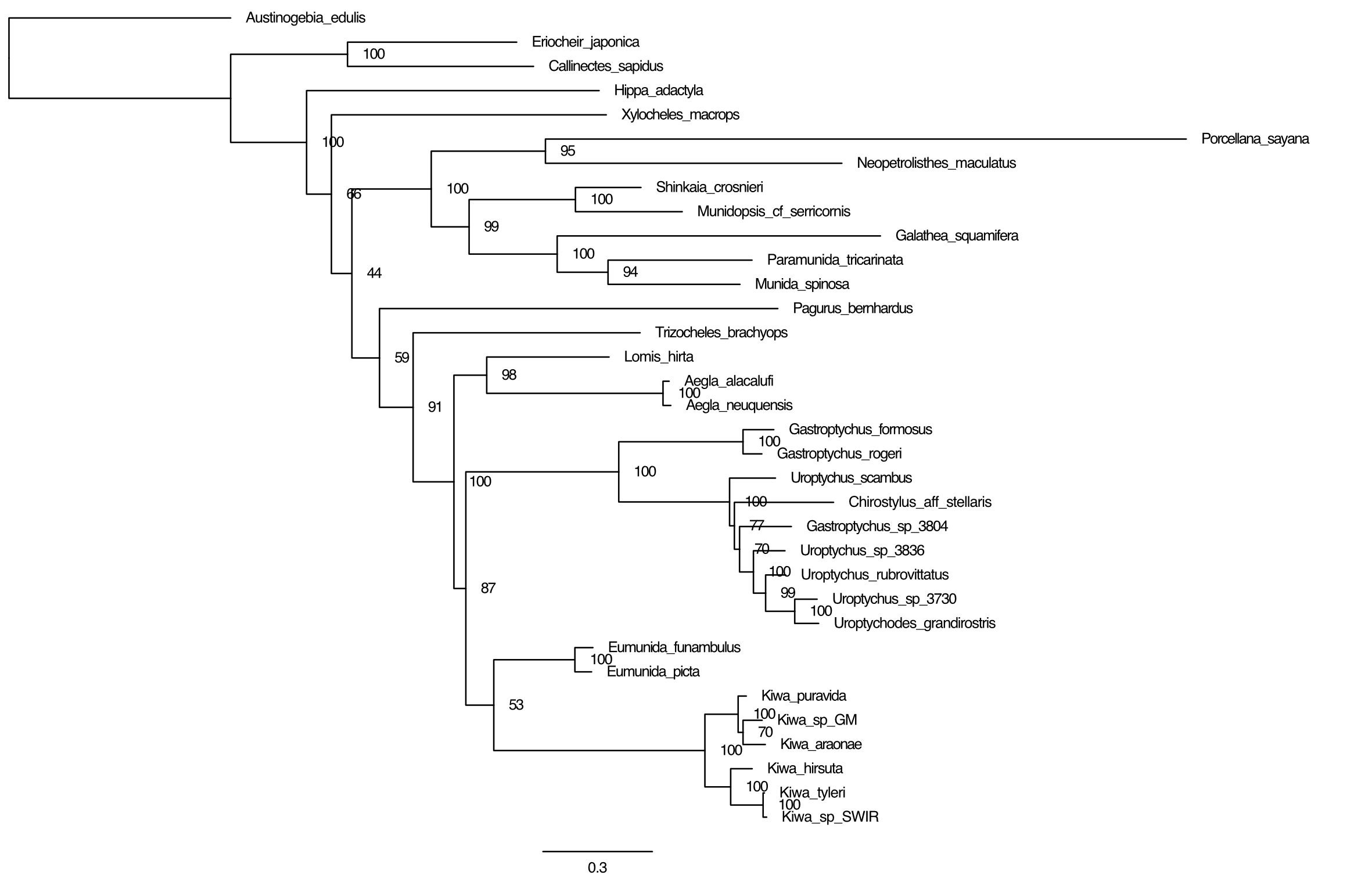

Supplement: S1 Fig — Node support numbers represent ML bootstrap percentages from 1000 non-parametric bootstrap replicates. (TIFF) [file pone.0194696.s008.tiff]

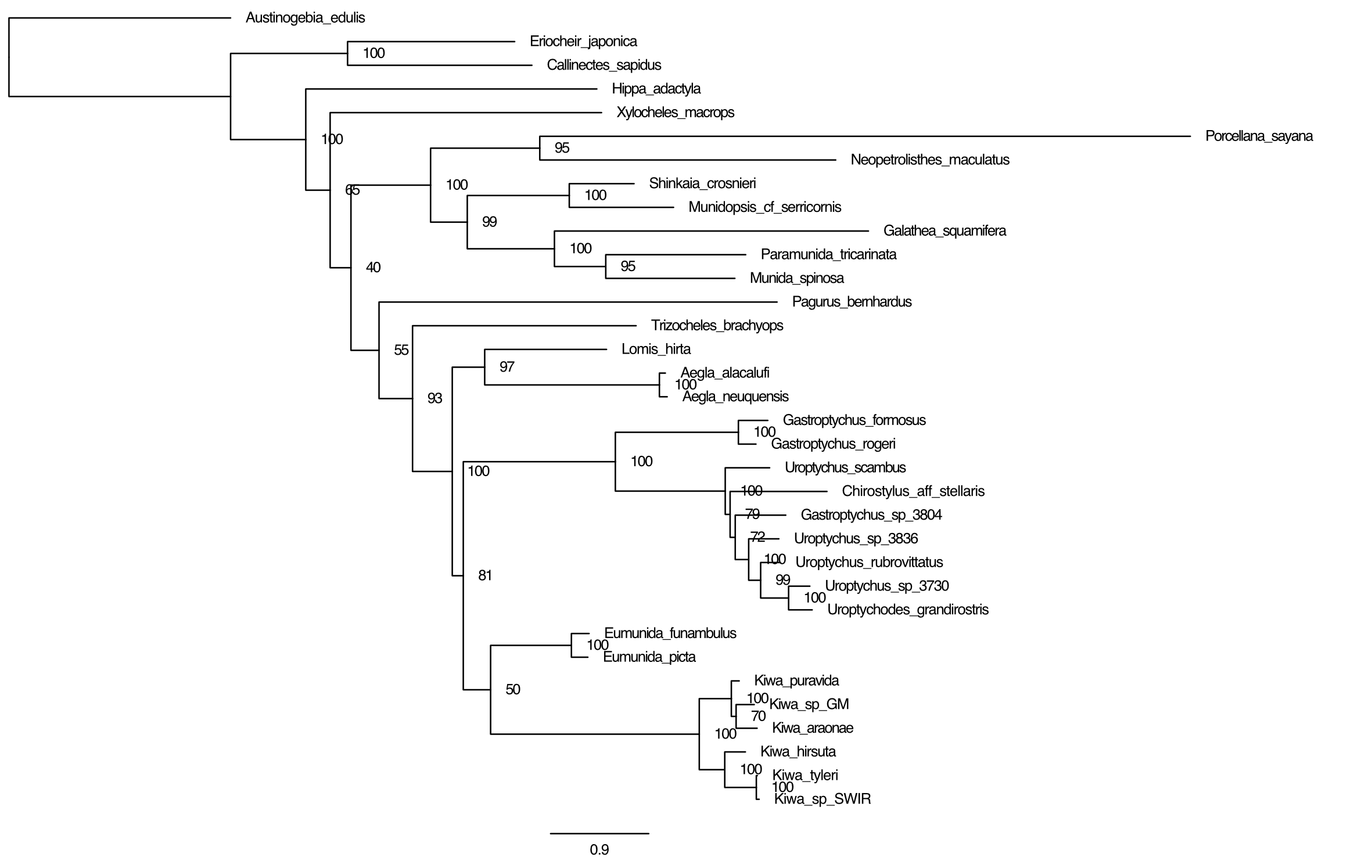

Supplement: S2 Fig — Node support numbers represent ML bootstrap percentages from 1000 non-parametric bootstrap replicates. (TIFF) [file pone.0194696.s009.tiff]

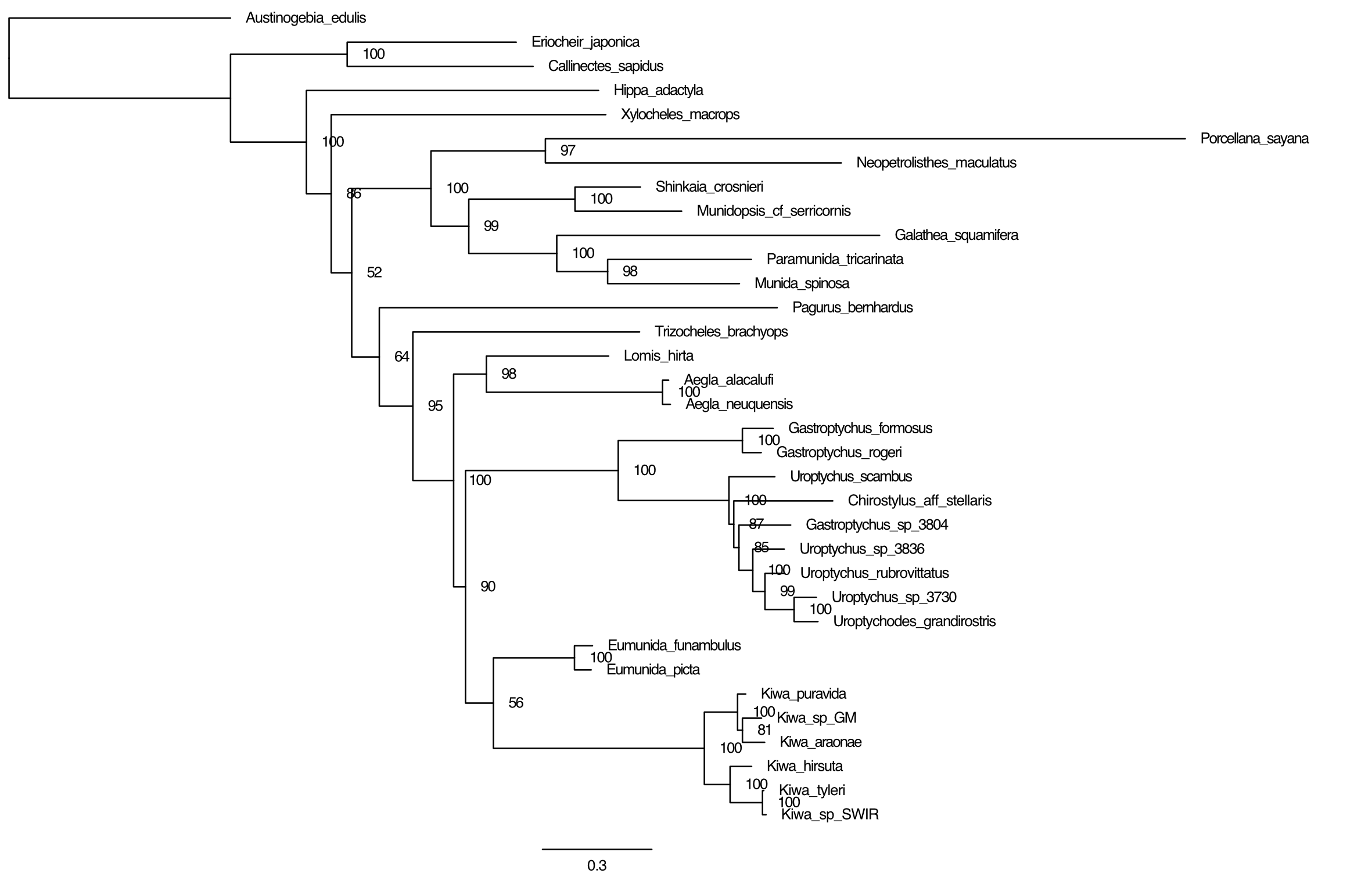

Supplement: S3 Fig — Node support numbers represent ultrafast approximate bootstrap percentages from 100,000 replicates. (TIFF) [file pone.0194696.s010.tiff]

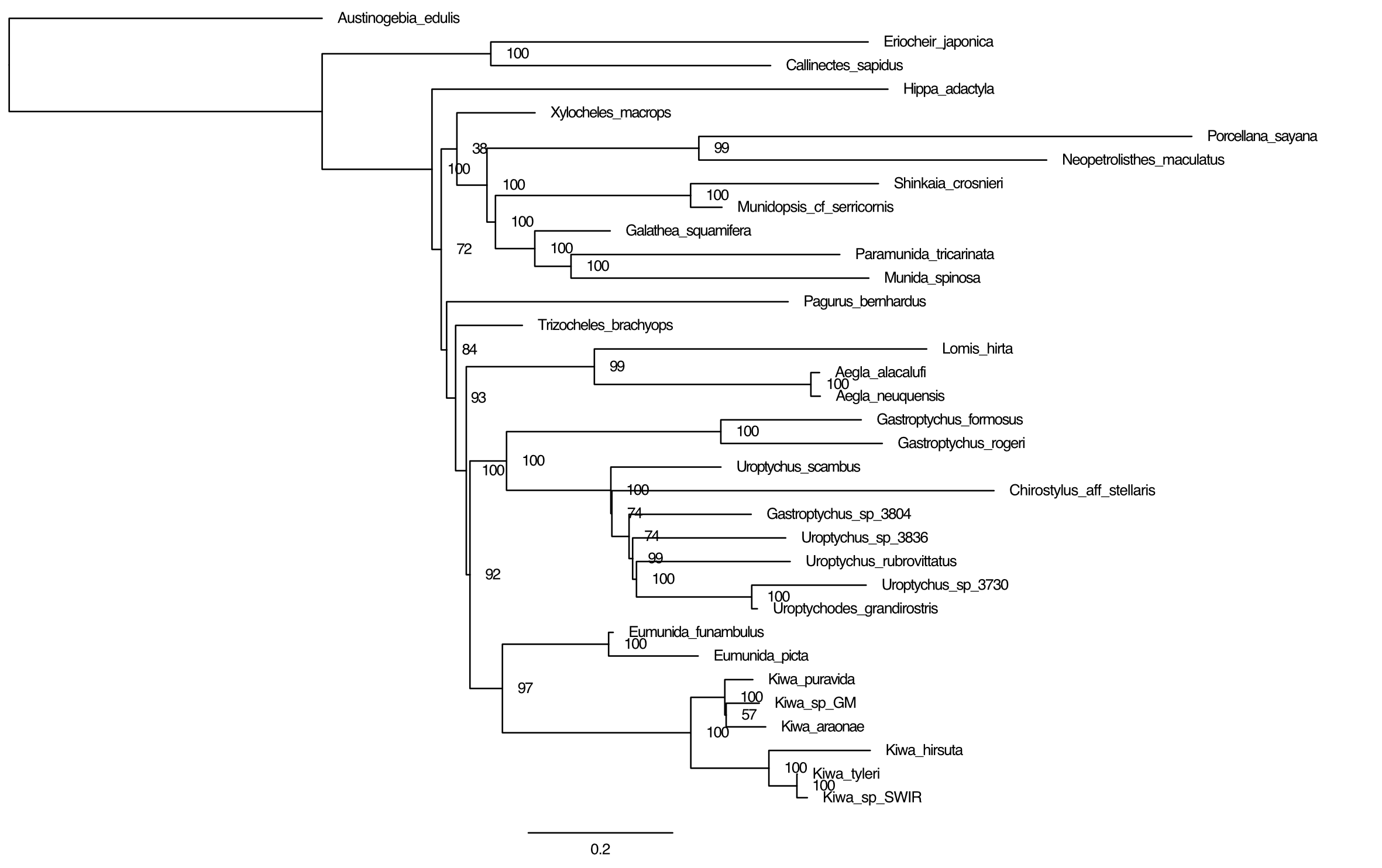

Supplement: S4 Fig — Node support numbers represent ultrafast approximate bootstrap percentages from 100,000 replicates. (TIFF) [file pone.0194696.s011.tiff]
